# Cas1 and Fen1 Display Equivalent Functions During Archaeal DNA Repair

**DOI:** 10.3389/fmicb.2022.822304

**Published:** 2022-04-15

**Authors:** Julia Wörtz, Victoria Smith, Jörg Fallmann, Sabine König, Tharani Thuraisingam, Paul Walther, Henning Urlaub, Peter F. Stadler, Thorsten Allers, Frank Hille, Anita Marchfelder

**Affiliations:** ^1^Biology II, Ulm University, Ulm, Germany; ^2^School of Life Sciences, University of Nottingham, Nottingham, United Kingdom; ^3^Department of Computer Science, Bioinformatics Group, Interdisciplinary Center for Bioinformatics, University of Leipzig, Leipzig, Germany; ^4^Bioanalytical Mass Spectrometry Group, Max Planck Institute for Multidisciplinary Sciences, Göttingen, Germany; ^5^Institute of Clinical Chemistry, University Medical Center Göttingen, Göttingen, Germany; ^6^Central Facility for Electron Microscopy, Ulm University, Ulm, Germany; ^7^German Centre for Integrative Biodiversity Research (iDiv) Halle-Jena-Leipzig, Leipzig, Germany; ^8^Competence Center for Scalable Data Services and Solutions, Leipzig Research Center for Civilization Diseases, University Leipzig, Leipzig, Germany; ^9^Facultad de Ciencias, Universidad Nacional de Colombia, Bogotá, Colombia; ^10^Institute for Theoretical Chemistry, University of Vienna, Vienna, Austria; ^11^Center for RNA in Technology and Health, University of Copenhagen, Copenhagen, Denmark; ^12^Santa Fe Institute, Santa Fe, NM, United States; ^13^Max Planck Institute for Mathematics in the Sciences, Leipzig, Germany

**Keywords:** CRISPR-Cas, Cas1, DNA repair, Fen1, archaea, *Haloferax volcanii*

## Abstract

CRISPR-Cas constitutes an adaptive prokaryotic defence system against invasive nucleic acids like viruses and plasmids. Beyond their role in immunity, CRISPR-Cas systems have been shown to closely interact with components of cellular DNA repair pathways, either by regulating their expression or via direct protein-protein contact and enzymatic activity. The integrase Cas1 is usually involved in the adaptation phase of CRISPR-Cas immunity but an additional role in cellular DNA repair pathways has been proposed previously. Here, we analysed the capacity of an archaeal Cas1 from *Haloferax volcanii* to act upon DNA damage induced by oxidative stress and found that a deletion of the *cas1* gene led to reduced survival rates following stress induction. In addition, our results indicate that Cas1 is directly involved in DNA repair as the enzymatically active site of the protein is crucial for growth under oxidative conditions. Based on biochemical assays, we propose a mechanism by which Cas1 plays a similar function to DNA repair protein Fen1 by cleaving branched intermediate structures. The present study broadens our understanding of the functional link between CRISPR-Cas immunity and DNA repair by demonstrating that Cas1 and Fen1 display equivalent roles during archaeal DNA damage repair.

## Introduction

Clustered regularly interspaced short palindromic repeats (CRISPR) and their associated genes (Cas) comprise a prokaryotic immune system, which defends bacteria and archaea from predatory mobile genetic elements, including viruses and plasmids (Hille et al., 2018; Nussenzweig and Marraffini, 2020). Its unique adaptive nature is enabled by proteins Cas1 and Cas2, which form a complex that integrates short pieces of invading DNA, called spacers, into the chromosomal CRISPR array (Barrangou et al., 2007; Nuñez et al., 2014). Transcription of the CRISPR array generates a long precursor CRISPR-RNA (crRNA), which is processed by Cas proteins or cellular RNases into short mature crRNAs, each harbouring the sequence of a previously acquired spacer (Brouns et al., 2008). Cas effector nucleases bind and utilise crRNAs to sequence-specifically target and cleave complementary sequences, causing the degradation of invading DNA, thus protecting the cell (Barrangou et al., 2007).

Besides their role in prokaryotic immunity, the involvement of CRISPR-Cas systems in cellular functions beyond anti-viral defence has been subject of multiple studies (Faure et al., 2019a,b). One such function is the contribution to the repair of chromosomal DNA damage. In fact, CRISPR-Cas systems were first hypothesised to represent a novel DNA repair system due to similarity of enzymatic domains in Cas proteins with those found in DNA repair proteins like DNA helicases and nucleases such as RecB (Makarova et al., 2002, 2006). While this assumption has now been revised, a functional link between components of DNA repair pathways and CRISPR-Cas systems has been reported previously; during spacer acquisition, the DNA repair complexes RecBCD and AddAB (in Gram-negative and Gram-positive bacteria, respectively) are able to degrade invading DNA, thereby providing Cas1-Cas2 with DNA fragments for integration (Marraffini and Sontheimer, 2008). This mechanism biases the uptake of new spacers toward foreign genomes, thus avoiding auto-immunity (Levy et al., 2015; Modell et al., 2017). Moreover, the integration of a new spacer generates temporarily single-stranded regions within the CRISPR array. Sealing of those gaps by repair proteins like DNA polymerases and ligases are crucial to retain chromosome integrity (Ivančić-Baće et al., 2015).

There are also indications that Cas proteins may actively participate in DNA damage repair. Deletion of *cas1* in *Escherichia coli* yielded a phenotype that was sensitive to DNA damage and showed impaired chromosome segregation. The mechanism behind these observations likely relies on the ability of Cas1 to cleave branched DNA substrates that usually occur during DNA repair and recombination (Babu et al., 2011; Rollie et al., 2015). Moreover, Cas1 has been shown to associate with various repair proteins in *E. coli*, further strengthening the assumption that components of the *E. coli* CRISPR-Cas system are involved in DNA repair (Babu et al., 2011).

Another example illustrating the interplay of the CRISPR immune system and DNA repair was revealed in *Sulfolobus solfataricus*. Here, the CRISPR-associated protein Csa3a controls the expression of major CRISPR adaptation genes (Liu et al., 2015), is a key player in the activation of DNA repair genes (Liu et al., 2017), and regulates the DNA damage response (DDR) (Liu et al., 2020). The reason for the synergistic activation of both CRISPR-Cas and DNA repair pathways by Csa3a is probably due to the frequent acquisition of spacers from its own genome (roughly 7%) (Liu et al., 2017). The simultaneous activation of DDR genes thus reduces auto-immunity effects caused by self-targeting spacers (Liu et al., 2020).

The recognition of similar DNA substrates by Cas and repair nucleases might explain the mutually exclusive prevalence of type II-A CRISPR-Cas systems and NHEJ (non-homologous end joining) genes. Csn2, an essential adaptation protein of type II-A systems (Heler et al., 2015; Wei et al., 2015), likely inhibits NHEJ repair by binding DNA at double-stranded breaks (DSBs) (Arslan et al., 2013), outcompeting the repair machinery and forcing bacteria to select against either of those systems (Bernheim et al., 2017).

In this study, we describe the interplay between CRISPR-Cas and DNA repair in the archaeon *Haloferax volcanii* by showing that Cas1 and repair protein Fen1 display equivalent roles during DNA repair. Fen1 is a highly conserved component of the cellular DNA metabolism found in Archaea and Eukarya that targets and removes 5’ flap structures commonly associated with Okazaki fragments or base excision repair (Pan et al., 2011; Balakrishnan and Bambara, 2013; Grasso and Tell, 2014). *Haloferax volcanii* has a single *fen1* gene located on the main chromosome (Lestini et al., 2010) while *cas1* is encoded on a megaplasmid within a type I-B CRISPR-Cas system (Maier et al., 2019). The *cas* gene cassette is flanked by two CRISPR loci, with a third CRISPR locus present on the main chromosome (Maier et al., 2015).

We found that deletions of either *cas1* or *fen1* caused similar phenotypes that exhibited sensitivity to oxidative stress and DNA damage. Double deletions caused cell elongation as well as increased cell size and when exposed to ultraviolet light (UV) stress survival rates were greatly reduced. We hypothesise that, given the similar substrate specificity and enzymatic activities of Cas1 and Fen1, both proteins are able to process 5′ flap secondary DNA structures that occur during DNA repair and DNA replication.

## Results

### Cas1 and Fen1 Are Required for DNA Repair

**FIGURE 1:**
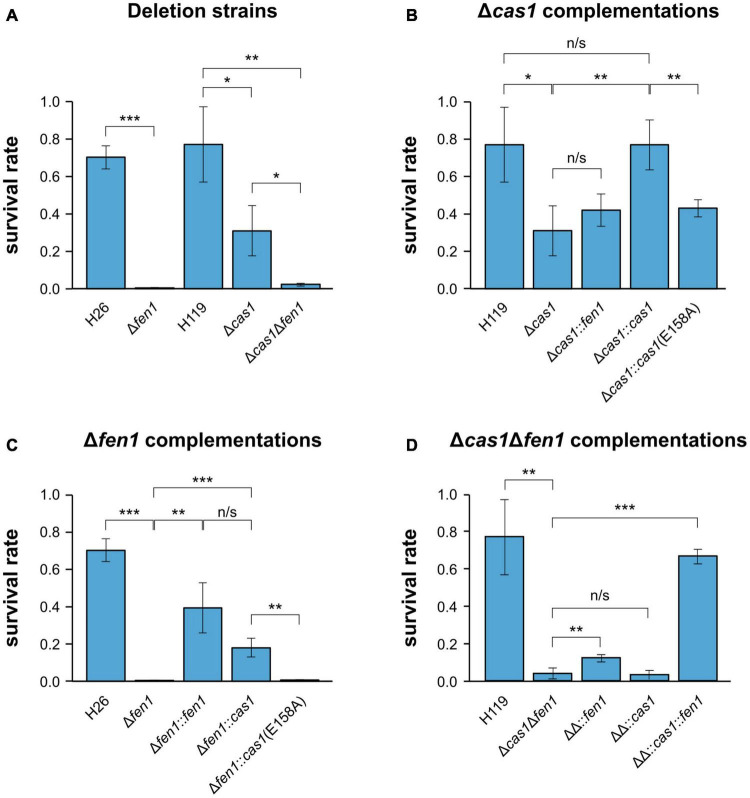
Survival rates of *Haloferax volcanii* exposed to 4 mM H_2_O_2_. *Haloferax volcanii* strains were grown to the mid-log growth phase, exposed for 1 h to 4 mM H_2_O_2_ and survival rates were calculated by dividing the number of colony forming units (CFUs) determined for the exposed strains by the number of CFUs of an unexposed control. **(A)** Wild-type and single deletion strains as well as the double deletion strain were analysed. H26 is the wild-type strain for Δ*fen1*, H119 is the wild-type strain for Δ*cas1* and Δc*as1*Δ*fen1* (Supplementary Table 4). **(B)** Δ*cas1* was complemented with *fen1*, *cas1* or a catalytically dead *cas1* mutant [*cas1(E158A)]*. **(C)** Δ*fen1* was complemented with *fen1*, *cas1* or the *cas1* mutant. **(D)** Δ*cas1*Δ*fen1* (ΔΔ) was complemented with *fen1*, *cas1* or the *cas1* mutant. Asterisks indicate significant differences (*t*-test) between the survival rates based on three independent experiments; ***: highly significant (p-value < 0.001), ** very significant (p-value < 0.01), * significant (p-value < 0.05), n/s = not significant.

In order to investigate whether there is a functional link between components of the CRISPR-Cas system and the DNA repair machinery, we induced DNA damage in the *H. volcanii* wild-type strains and mutant daughter strains, harbouring a deletion of either *cas1, fen1* or both genes, respectively, and evaluated their fitness. To evaluate DNA repair, all strains were exposed to oxidative stress using H_2_O_2_ and survival rates were measured. Both single deletion strains displayed a significant reduction in survival compared to the wild-type, with Δ*fen1* showing a higher sensitivity than Δ*cas1* (Figure 1A). The Δ*cas1*Δ*fen1* double mutant displayed a growth defect comparable to the Δ*fen1* single mutant. These results indicate that both Cas1 and Fen1 are involved in the cellular oxidative stress response.

To exclude any polar effects of the gene deletions, we complemented the Δ*cas1* strain with a plasmid-born copy of the gene and were able to detect growth rescue in the complemented strain (Figure 1B). Moreover, when mutating the active site of Cas1 in the complementing gene (E158A, Supplementary Figure 1) (Babu et al., 2011; Kim et al., 2013; Rollie et al., 2015), growth rescue was impaired, further strengthening our assumption that the enzymatic activity of Cas1 is directly involved in DNA repair (Figure 1B).

We also supplemented the Δ*cas1* strain with a plasmid-borne copy of *fen1* but were not able to observe increased rescue, indicating that the DNA damage induced by H_2_O_2_ cannot be compensated by higher levels of Fen1 (Figure 1B). Previous reports showed that Cas1 has not only a 5′ flap processing activity but additional activities (Han et al., 2009; Wiedenheft et al., 2009; Beloglazova et al., 2015), which might explain why Fen1 cannot completely rescue a missing Cas1. Interestingly, when we overexpressed Cas1 in the Δ*fen1* background, survival rates increased compared to both the uncomplemented Δ*fen1* strain and the Δ*fen1* strain harbouring a plasmid expressed active-site mutant of *cas1* (Figure 1C), clearly demonstrating the capacity of Cas1 to mitigate DNA damage. It is noteworthy that the Cas1 protein is only present in low levels in wild-type cells under standard conditions (Jevtić et al., 2019), which could explain why the overexpression of Cas1 showed a significant increase in cell survival, as opposed to Fen1 in the Δ*cas1* strain.

In the Δ*cas1*Δ*fen1* background, complementation with only *cas1* did not significantly increase the survival rate, while complementation with only *fen1* showed a slight but statistically significant growth rescue (p-value: 1,46E-02) (Figure 1D). These data are consistent with the results of the single deletion strains, where Δ*fen1* showed an elevated sensitivity toward oxidative stress (p-value: 3.47E-06) compared to Δ*cas1* (p-value: 1.68E-02).

**FIGURE 2:**
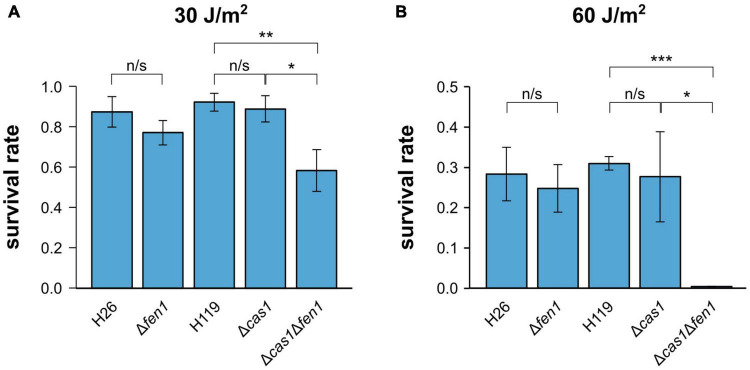
Survival rates of *Haloferax volcanii* exposed to UV radiation. *H. volcanii* strains were grown to the mid-log growth phase, spotted on Hv-YPC plates and exposed to UV radiation [**(A)** 30 J/m^2^, **(B)** 60 J/m^2^]. After growth, survival rates were calculated by dividing the number of CFUs determined for the exposed strains by the number of CFUs of an unexposed control. Asterisks indicate significant differences (*t*-test) between the survival rates based on three independent experiments; ***: highly significant (p-value < 0.001), ** very significant (p-value < 0.01), * significant (p-value < 0.05), n/s = not significant.

To further evaluate the ability of Cas1 and Fen1 to repair DNA damage, we exposed each single deletion strain and the double mutant to UV radiation (Figure 2). We observed a significant decrease in cell survival at both 30 and 60 J m^–2^, whereby only 1% of Δ*cas1*Δ*fen1* cells survived at 60 J m^–2^ (Figure 2B). Interestingly, the single mutants did not display an increased sensitivity to UV radiation, indicating that UV-induced damage can be efficiently repaired in the presence of either enzyme alone.

### Absence of Cas1 and Fen1 Cause Increased Cell Size and Cell Elongation

Our results indicate that both Cas1 and Fen1 play vital roles during the DNA damage response. To gain further insights into their roles in cellular growth, we compared morphological features of the wild-type and the Δ*cas1*Δ*fen1* deletion strain.

**FIGURE 3:**
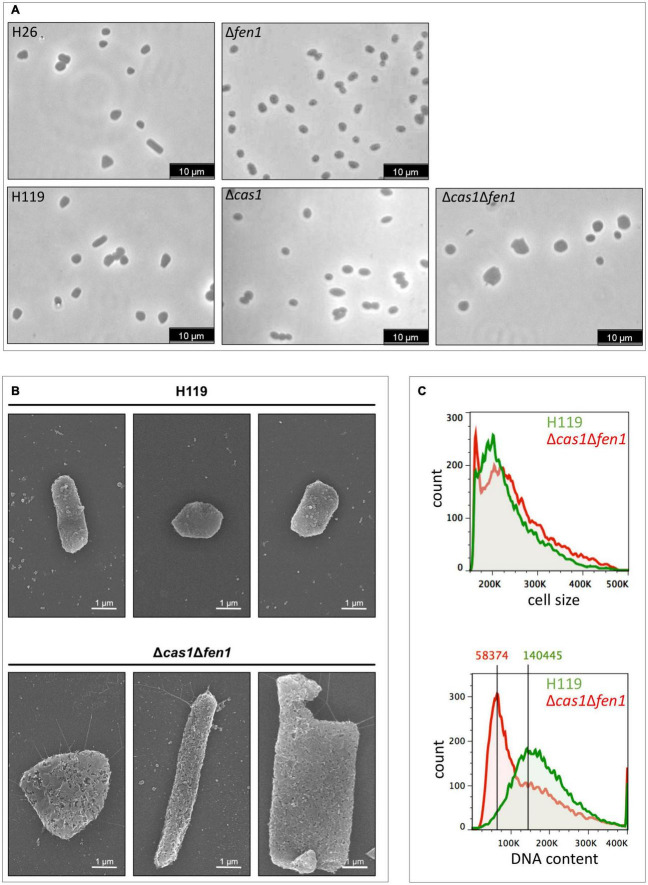
Cell morphology and DNA content of wild-type and the Δ*cas1*Δ*fen1* deletion strain. **(A)** *Haloferax volcanii* wild-type and deletion strains were grown to mid-log growth phase and cell morphology was analysed by light microscopy. **(B)** Scanning electron microscopic pictures of wild-type strain H119 (upper panel) and Δ*cas1*Δ*fen1* (lower panel) grown to stationary phase. **(C)** Flow cytometry of wild-type H119 (green) and Δ*cas1*Δ*fen1* deletion strains (red) grown to mid-log growth phase. The FS (forward light scatter) channel was used to evaluate cell size (upper panel) and the FL1 (fluorescence 1) channel was used to evaluate the DNA content of acridine orange-stained cells (lower panel). Modal population values for DNA content highlights a decreased DNA content in the Δ*cas1*Δ*fen1* mutant (41.5% of wild-type value).

Analysis of the strains via light microscopy revealed that the double mutant displayed an inflated phenotype under normal growth conditions, while the single deletion strains (Δ*cas1* and Δ*fen1*, respectively) lacked any obvious change in phenotype (Figure 3A), indicating that the deletion of both genes has effects on cellular pathways other than DNA damage response. The altered morphology of the double mutant was confirmed by scanning electron microscopy (Figure 3B). Here, the cells showed a variety of elongated and inflated shapes, indicating defects in the growth cycle and division of the double mutant strain. This assumption was further strengthened by analysing cell size and DNA content using flow cytometry (Figure 3C and Supplementary Figure 2). Here, we observed a shift of the peak absorbance of the DNA content in the Δ*cas1*Δ*fen1* strain compared to the wild-type, indicating fewer genome copies per cell in the mutant. Considering the enlarged phenotype of the mutant, these results suggest that the mutant fails to properly replicate its genome, leading to impaired cell division, resulting in the cell inflating. It can therefore be hypothesised that Cas1 and Fen1 may play a role in DNA-related pathways, such as DNA replication, to maintain genome integrity not only during stress responses but under normal growth conditions as well.

**FIGURE 4:**
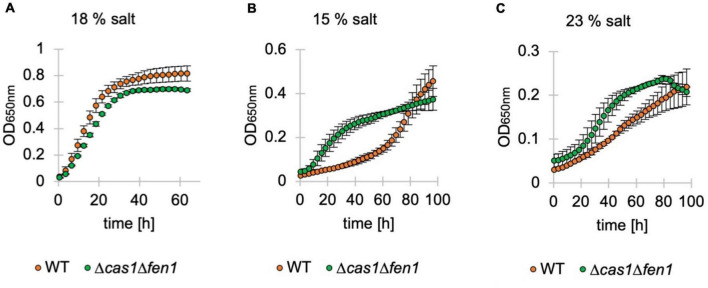
Growth of deletion mutants in standard, low and high-salt conditions. Growth of *Haloferax volcanii* wild-type (orange) and deletion strains (green) under standard (18%) **(A)**, low (15%) **(B)** and high (23%) **(C)** salt conditions in Hv-YPC medium. Vertical lines represent standard deviation at different measuring points of three independent experiments. The x-axis shows the time of growth and the y-axis the OD_650nm_.

To assess whether the inflated phenotype of the Δ*cas1*Δ*fen1* mutant affects its ability to deal with osmotic stress, we monitored the growth of *H. volcanii* in media supplemented with different salt concentrations^1^ via OD_650nm_ measurements (Figure 4). Under standard conditions (18% salt), we observed a similar growth rate of the wild-type and double deletion strain (Figure 4A).

Interestingly, in both low and high salt conditions (15% and 23% salt, respectively), the mutant displayed a shorter lag phase compared to the wild-type, indicating an increased adaptation rate to osmotic stress in the absence of Cas1 and Fen1 (Figures 4B,C). However, when grown at 23% salt, the optical density of the double mutant culture declined shortly after reaching the stationary phase at roughly 80 h post-inoculation, while the wild-type was still in the logarithmic growth phase at a similar timepoint.

### Cas1 Interacts With DNA Repair Proteins

To exclude the possibility that the deletion of *cas1* and *fen1* has an effect on the expression level of other DNA repair proteins, thus indirectly causing impaired fitness during the cellular DNA damage response, we investigated gene expression patterns in both the wild-type and Δ*cas1*Δ*fen1* deletion strains using RNA sequencing. When comparing up- and down-regulated genes (Tables 1, 2 and Supplementary Tables 1, 2), only minor changes were observed in the gene expression levels of various functional groups that are not involved in DNA damage repair or DNA metabolism. However, the absence of Cas1 and Fen1 caused the up-regulation of altogether 15 putative proviral genes (Dyall-Smith et al., 2021) in the genome of *H. volcanii* (Table 1 and Supplementary Table 1), which might be relevant for the immunity-related functions of Cas1.

**TABLE 1:** RNA expression profile of the Δ*cas1*Δ*fen1* deletion mutant compared to the wild-type.

Gene_ID	Annotation/Gene name	logFC
**Provirus regions**		
HVO_0369	hypothetical protein; ProVir2 prediction	2.18
HVO_1434	hypothetical protein; ProVir5 prediction	2.16
HVO_A0218	oxidoreductase; ProVir4 prediction	2.01
HVO_0276A	homolog to HGPV1-ORF9; ProVir6 prediction	2.00
**Iron metabolism**		
HVO_1721	2Fe-2S iron-sulfur cluster binding domain-containing protein, *ferA3*	2.04
**Transposases**		
HVO_2817	transposase (ISH51)	2.06
HVO_A0258	ISH4-type transposase homolog	2.06
**Other**		
HVO_B0028	xylose dehydrogenase (NAD/NADP dependent), *xacA*	2.32
HVO_B0342	oxidoreductase (Luciferase family protein), *mer2*	2.19
HVO_0641	cob(II)yrinic acid a,c-diamide reductase, *bluB*	2.10
HVO_B0343	hydrolytic enzyme LplD, *lplD*	2.07
HVO_0694	purine phosphoribosyltransferase, *gptA*	2.05
HVO_1205	taxis cluster protein CheD, *cheD*	2.00

*Shown are up-regulated genes (logFC ≥ 2.00) (logFC: log_2_ fold change.). Six genes for hypothetical proteins are also up-regulated with logFC ≥ 2.00 (Supplementary Table 1), for a complete list of up-regulated genes see Supplementary Table 1. Provirus prediction according to Dyall-Smith et al. (2021).*

**TABLE 2:** RNA expression profile of the Δ*cas1*Δ*fen1* deletion mutant compared to the wild-type.

Gene_ID	Annotation/Gene name	logFC
**Deleted genes**		
HVO_2873	flap endonuclease, *fen1*	−8.13
HVO_A0211	Cas1 protein, *cas1*	−7.63
**Transposases**		
HVO_A0279	Transposase (ISH18)	−4.41
HVO_2051	Transposase (ISH51)	−2.23
**Transcription regulators**		
HVO_2507	Asn family transcription regulator, *trh7*	−3.17
HVO_2522	Asn family transcriptional regulator, *trh8*	−2.97
**tRNA metabolism**		
HVO_1092	ribonuclease P protein component 2, *rnp2*	−2.68
HVO_3052	tRNA Gly	−2.35
**Iron metabolism**		
HVO_A0541	ABC-type transport system periplasmic substrate-binding protein (probable substrate iron-III)	−2.45
HVO_B0044	iucA iron transport protein A, *iucA*	−2.43
HVO_2588	isocitrate dehydrogenase, *iucD*	−2.28
**Other**		
HVO_1228	halocyanin domain protein (membrane), *hcpE*	−2.92
HVO_2508	carbamoyl-phosphate synthase small subunit, *carA*	−2.56
HVO_2361	carbamoyl-phosphate synthase large subunit, *carB*	−2.04
HVO_B0045	daminobutyrate decarboxylase, *bdb*	−2.47
HVO_B0046	diaminobutyrate pyruvate aminotransferase, *dat*	−2.26
HVO_2606	PQQ repeat-containing protein	−2.72
HVO_2607	PQQ repeat-containing protein	−2.29

*Shown are down-regulated genes (logFC ≤ 2.00) (logFC:log2 fold change.). Nine genes for hypothetical proteins are also down-regulated with logFC ≤ 2.00 (Supplementary Table 2), for a complete table of down-regulated genes see Supplementary Table 2.*

Considering the minor changes in observed expression patterns, we conclude that the deletion of *cas1* and *fen1* does not strongly impact cellular pathways on a transcriptional level.

We further analysed direct protein-protein interactions of Cas1 by conducting pull-down analyses. Cas1 co-purified with a variety of proteins involved in replication and DNA repair (Table 3), including several helicases and the DNA mismatch repair protein MutS, and was associated with three members of the UvrABC system that acts in nucleotide excision repair. For a final confirmation of these interactions reverse pull-down experiments have to be carried out where the proteins identified by the Cas1 pull-down are FLAG-tagged and the presence of Cas1 in the elution fraction is determined after FLAG-purification.

**TABLE 3:** Proteins that were co-purified with Cas1.

Gene ID	Annotation/Gene name	Peptides/Unique spectra counts
**Replication and repair**		
HVO_0393	UvrABC system protein A, *uvrA*	73
HVO_0029	UvrABC system protein B, *uvrB*	32
HVO_0415	repair helicase UvrD, *uvrD*	38
HVO_0349	DNA-directed RNA polymerase subunit A, *rpoA1*	56
HVO_0347	DNA-directed RNA polymerase subunit B, *rpoB2*	44
HVO_0858	DNA-directed DNA polymerase B (intein-containing), *polB1*	36
HVO_2380	AAA-type ATPase (CDC48 subfamily), *cdc48a*	66
HVO_0854	DNA double-strand break repair ATPase Rad50, *rad50*	56
HVO_B0118	Smc-like protein Sph2; homolog of Rad50, *sph2*	30
HVO_0552	DNA mismatch repair protein MutS, *muts1b*	49
HVO_0014	ATP-dependent DNA helicase Hel308a, *hel308a*	44
HVO_1018	Hef-associated 3 exonuclease, *recJ3*	35
HVO_2889	DHH/RecJ family phosphoesterase RecJ4, *recJ4*	33
HVO_0220	ATP-dependent DNA helicase MCM, *mcm*	34
**Cas protein**		
HVO_A0206	Cas8b, *cas8*	58
**Ribonucleases**		
HVO_0874	zinc-dependent nuclease CPSF1, *cpsf1*	44
HVO_2724	ribonuclease J, *rnJ*	35
**Sensing kinases**		
HVO_1811	sensor box histidine kinase	32
HVO_B0154	receiver/sensor box histidine kinase	41

*Two independent pull-down analyses using a FLAG-Cas1 fusion protein resulted in the co-purification of interacting proteins. Corresponding peptides of the co-purifying proteins were sequenced by mass spectrometry (MS). Only proteins that are functionally interesting, e.g., involved in DNA repair, CRISPR-Cas functions, and having unique spectra/peptide counts ≥ 30 are listed. The complete table of co-purified proteins with a spectra/peptide count ≥ 30 is found in Supplementary Table 3. The complete set of MS data including all co-purified proteins (including those with a spectra/peptide count < 30) has been deposited in PRIDE. Column peptides/unique spectra counts: number of peptides and unique spectra counts identified.*

Other proteins that co-purified with Cas1 included RNases, sensing kinases, and proteins associated with amino acid synthesis, vitamin and glucose metabolism (Table 3 and Supplementary Table 3). Interestingly, only five peptides of Fen1 were detected during the MS analysis (data not shown, the complete set of co-purified proteins has been deposited in PRIDE), suggesting that the interaction between Cas1 and Fen1 is only weak or does not occur at all. This indicates that their functional link might not be based on physical interactions within the cell.

### Cas1 Has 5′ Flap Endonuclease Activity

**FIGURE 5:**
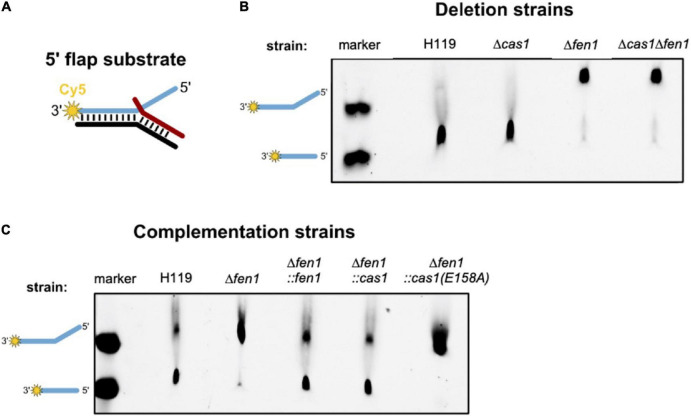
*In vitro* processing of a 5′ flap DNA substrate. Cell extracts of *Haloferax volcanii* wild-type and deletion strains were prepared and incubated with a 5′ flap DNA substrate. **(A)** Representation of the 5′ flap substrate, which was labelled with Cy5 (yellow star) at the 3′ end of the flapped (+) strand. **(B)** Denaturing PAGE showing the DNA processing activity of cell extracts of the deletion strains. Lane marker: full length Cy5 labelled oligonucleotide as shown in A and Cy5 labelled oligonucleotide corresponding to the processed product; lanes H119, Δ*cas1*, Δ*fen1*, Δ*cas1*Δ*fen1*: soluble extracts from respective strains. **(C)** Denaturing PAGE showing the processing activity of cell extracts of the deletion strains complemented with the genes for Fen1, Cas1 or the Cas1 mutant. Shown are representative gels of three independent experiments.

Previous studies have revealed that Cas1 has DNA processing activities and is able to cleave branched DNA substrates such as Holliday junctions and 5′ flap structures (Babu et al., 2011; Rollie et al., 2015). We hypothesised that the type I-B Cas1 homolog of *H. volcanii* might possess similar activities on branched DNA substrates. In the context of DNA repair, processing of such structures by Cas1 might compensate for a deletion of the flap endonuclease Fen1. To test this, we attempted to express and purify both nucleases recombinantly in *E. coli* but failed to achieve adequate yield and purity. Therefore, we prepared cell extracts of *H. volcanii* wild-type as well as *cas1* and *fen1* deletion mutants and compared their ability to process a double flap DNA substrate (Figure 5).

We observed processing of the branched substrate by cell extracts of the wild-type and Δ*cas1* strain, whereas cell extracts of the Δ*fen1* strain yielded no DNA cleavage product, suggesting that only Fen1 is able to process the DNA double flap substrate (Figure 5B). It is noteworthy that Cas1 levels are considerably low under normal growth conditions (Jevtić et al., 2019), potentially too low to observe substrate cleavage in our assay. Therefore, we repeated the cleavage assay using cell extracts of a Δ*fen1* strain that constitutively expressed an *in trans* copy of *cas1* and were able to detect processing of the double flap substrate (Figure 5C). Moreover, this effect was not observable in the Δ*fen1* strain expressing a nuclease-defective variant of Cas1 (E158A), which strongly indicates that both Fen1 and Cas1 are able to recognise and process a DNA flap.

## Discussion

Thus far it has only been demonstrated in bacteria that components of the prokaryotic immune system CRISPR-Cas may be involved in other cellular pathways, such as DNA repair. In this study, we demonstrate a functional link between CRISPR-Cas systems and DNA metabolism in Archaea, by showing that Cas1 and the replication enzyme Fen1 play equivalent roles during the DNA damage response in *H. volcanii*.

Fen1 is found in Archaea and Eukarya and cleaves flapped DNA structures that occur during DNA replication and repair (Pan et al., 2011; Balakrishnan and Bambara, 2013). Accordingly, deletion of the homologous gene in *H. volcanii* causes reduced survival rates following DNA-damaging oxidative stress. Deletion of *cas1* had similarly adverse effects on cell survival under these conditions, indicating that the CRISPR-Cas immune protein is involved in DNA repair as well. Additionally, we found that the enzymatic activity of the Cas1 nuclease is directly responsible for DNA repair, as a mutation in the active site of the protein also resulted in reduced survival after oxidative stress. The mechanistic basis for repairing DNA damage is likely linked to the ability of the nuclease Cas1 to cleave flapped DNA substrates in a similar manner to Fen1.

Pull-down experiments showed that Cas1 seems to physically interact with a variety of DNA binding and processing enzymes, some of which are involved in DNA repair. Whether these interactions affect the role of Cas1 in DNA repair remains to be revealed, however, it is tempting to speculate that Cas1 is part of a multi-protein repair complex in *H. volcanii*. The formation of such a complex might determine the role of Cas1 in either DNA repair or CRISPR-Cas immunity, which would be advantageous for the cell. During viral defence, Cas1 forms a complex with Cas2 and integrates short pieces of DNA into the CRISPR array (Nuñez et al., 2014). Recruiting the Cas1-Cas2 complex to DNA lesions during repair could trigger spacer acquisition from the organism’s own genome and consequently lead to auto-immunity. The interaction of Cas1 with DNA repair enzymes would prevent this problem, since complex formation with Cas2 is constrained and thus prevents spacer acquisition. Further experiments are required to reveal the factors that influence the role of Cas1 in either damage repair or immunity. In our pull-down experiments Cas2 could not be identified as interaction partner of Cas1. Cas2 is not detectable in proteome analyses of cells grown under standard conditions (Jevtić et al., 2019; Schulze et al., 2020) which might explain this observation.

In addition to its role in DNA repair, Cas1 might also play a role in DNA replication. In *E. coli*, it has been proposed that Cas1 is involved in genome segregation, based on the detection of abnormally elongated cells of a *cas1* knockout strain after mitomycin C treatment (Babu et al., 2011). In *H. volcanii*, a single deletion of either *fen1* or *cas1* did not cause any notable morphological changes in cell shape or size, compared to the wild-type under unstressed conditions. However, a simultaneous deletion of both genes led to an inflated phenotype and reduced DNA content per cell, with the latter being indicative of fewer genome copies per cell. Similar morphological alterations have been observed before, where UV-induced DNA damage in *H. volcanii* results in increased cell size and cell elongation (Delmas et al., 2013). These results suggest that both Cas1 and Fen1 might process flapped DNA substrates during replication and thus maintain genome integrity under conditions of genomic stress.

## Conclusion

This study expands our knowledge of the role of CRISPR-Cas systems beyond immunity, by demonstrating that, similar to its bacterial homologs, the archaeal Cas1 in *H. volcanii* may participate in cellular DNA repair.

## Materials and Methods

Strains, plasmids and oligonucleotides used are listed in Supplementary Tables 4–6. *E. coli* strain DH5a (Invitrogen, Thermo Fischer Scientific, Waltham, MA, United States) was used for plasmid cloning and grown aerobically at 37°C in 2YT medium (Miller, 1972).

### Growth Curves

*Haloferax volcanii* strains were grown in Hv-YPC medium (Dyall-Smith, 2008) at 45°C with an optimal concentration of 18% salt water (2.46 M NaCl, 88 mM MgCl_2_, 85 mM MgSO_4_, 56 mM KCl, 12 mM Tris-HCl, pH 7.5). For low and high salt stress, salt concentration was adjusted to 15% and 23%, respectively, without modifying the ion ratios. While standard conditions (18% salt water) correspond to 15% NaCl, low salt (15% salt water) corresponds to 10.8% NaCl and high salt (23% salt water) corresponds to 19.2% NaCl. Growth of cultures was monitored in 96 well microtiter plates using an Epoch2 NS Microplate Spectrophotometer (BioTek Instruments, Bad Friedrichshall, Germany). Strains were precultured in Hv-YPC medium to OD_650nm_ of 0.4–0.7, after dilution to an OD_650nm_ of 0.05 they were transferred to microtiter plates. Cultures were incubated aerobically with orbital shaking at 45°C and OD_650nm_ was measured every 30 min. Outer wells were filled with salt water as evaporation barriers (Liao et al., 2016). The growth curves represent the average of at least three biological replicates.

### Plasmid Cloning

pBlue-*Nde*I-Cas1-*Eco*RV was generated by ligating a linearised pBluescriptII KS (cleaved with *Eco*RV) with the insert that contained *cas1* and the restriction sites *Nde*I and *Eco*RV. The insert was amplified from *H. volcanii* genomic DNA using primers Cas1up-FLAGC and Cas1do-FLAGC, primers contained the restriction sites *Nde*I and *Eco*RV.

pTA927-Cas1E158A-CFLAG was generated using a site-directed mutagenesis kit (Agilent, Santa Clara, CA, United States) according to the manufacturer’s instructions with primers Cas1E158A fw and Cas1E158A rev and pTA927-Cas1-CFLAG as a template.

Plasmid pTA927-FLAGcontrol was cloned as follows. Using primers FLAG *Nde*I fw and FLAG *Eco*RI rev with pTA927-NZ-NFLAG as template, the FLAG sequence (ATG-3xFLAG-TGA) was amplified. The primers contained restriction sites for *Apa*I (5′) and *Eco*RI (3′), after digestion of the resulting polymerase chain reaction (PCR) product with *Apa*I and *Eco*RI ligation with pTA927 (digested with *Apa*I and *Eco*RI) resulted in pTA927-FLAGcontrol.

Plasmid pTA231.p.fdx was obtained by ligation of pTA231 (digested with *Not*I and *Eco*RI) with a p.fdx-t.syn fragment excised with *Not*I and *Eco*RI from pMA-T-FdxSyn (synthesised by GeneArt, Thermofisher Scientific), resulting in pTA231-p.fdx.

In order to generate pTA231-p.fdx-NFLAG, a PCR with primers 231NFLAG fw and 231NFLAG rev and pTA927-Cas1-NFLAG as a template was performed resulting in PCR fragment ATG-3xFLAG, primers contained a *Nde*I restriction site (5′ terminus) and a *Sna*BI as well as a *Xba*I restriction site (3′ terminus). The purified PCR product was digested with *Nde*I and *Sna*BI and ligated to pTA231-p.fdx (digested with *Nde*I and *Xba*I), to generate pTA231-p.fdx-NFLAG.

Plasmid pTA230-p.fdx-NFLAG was generated by digesting pTA231-p.fdx-NFLAG with *Not*I and *Eco*RI to obtain the p.fdx-NFLAG-t.syn fragment (p.fdx-ATG-3x FLAG-t.syn), which was subsequently ligated with the pTA230 vector (linearised with *Not*I and *Eco*RI).

Plasmids pTA230-p.fdx-Cas1-NFLAG and pTA230-p.fdx-Fen 1-NFLAG were obtained as follows. *fen1* and *cas1* genes were amplified from *H. volcanii* genomic DNA using primers Fen1 *Sna*BI fw/Fen1 *Xba*I rev and Cas1 *Eco*RV fw/Cas1 *Xba*I rev, respectively. The resulting PCR products were ligated with pBluescriptII KS (*Eco*RV digested), resulting in pBlue-*Sna*BI-Fen1-*Xba*I and pBlue-*Eco*RV-Cas1-*Xba*I. These plasmids were then digested with *Sna*BI/*Xba*I or *Eco*RV/*Xba*I to excise the *fen1* and *cas1* gene fragments, respectively, which were subsequently ligated with pTA230-p.fdx-NFLAG (linearised with *Sna*BI and *Xba*I) yielding pTA230-p.fdx-Fen1-NFLAG and pTA230-p.fdx-Cas1-NFLAG.

For generation of pTA230-p.fdx-Cas1E158A-NFLAG, a site-directed mutagenesis kit (Agilent, Santa Clara, CA, United States) was used according to the manufacturer’s instructions with primers Cas1E158A fw and Cas1E158A rev and pTA230-p.fdx-Cas1-NFLAG as a template.

To obtain the pTA962-p.fdx-Fen1-NFLAG-Cas1 overexpression plasmid, the *cas1* insert was generated with PCR using primers 5′XmaJI Cas1 + Start fw/3′BglII Cas1 + Stopp rev and plasmid pTA230-p.fdx-Cas1-NFLAG as template. The resulting PCR product was cleaved with *Xma*JI/*Bgl*II and then ligated with pTA962 (cleaved with *Xma*JI/*Bgl*II). To generate p.fdx-Fen1-NFLAG, the *fen1* insert was obtain with PCR using primers 5′ApaI p.fdx fw/3′XmaJI Fen1 + Stopp rev and pTA230-p.fdx-Fen1-NFLAG as template. The resulting PCR product was cloned into pTA962 via *Apa*I/*Xma*JI restriction sites.

The *cas1* deletion plasmid pTA131-UPDO(*cas1*) was obtained by amplifying the *cas1* gene with 480 bp up- and 587 bp down-stream sequences using primers Cas1KOUP/Cas1KODO. The resulting fragment was ligated with pTA131 (linearised with *Eco*RV). The resulting plasmid pTA131-UP-Cas1-DO was used as template for an inverted PCR with 5′ phosphorylated primers iCas1 fw/iCas1 rev, the resulting PCR product was ligated to obtain pTA131-UPDO(*cas1*). This plasmid contains only the up- and downstream regions of *cas1*.

### Generation of Knockout Mutants

Deletion strains were generated using the pop-in pop-out method as described previously (Bitan-Banin et al., 2003). Transformations were performed according to the PEG600 protocol as described in the HaloHandbook (Dyall-Smith, 2008). Strain Δ*cas1* was generated as described (Klein, 2011), in short: For the generation of the Δ*cas1* knockout strain, the H119 wild-type strain was transformed with the *cas1* deletion plasmid pTA131-UPDO(*cas1*) and plated onto Hv-Ca medium (Dyall-Smith et al., 2021) supplemented with L-tryptophan (40 μg/ml) to select for pop-in candidates which were subsequently identified by PCR using the primers Cas1 KO UP/Cas1 KO DO. Positive clones were then grown in Hv-Ca medium supplemented with L-tryptophan (40 μg/ml) and subsequently plated onto Hv-Ca agar plates with L-tryptophan (40 μg/ml), 5-FOA (5-fluoro-orotic acid) (10 μg/ml) and 10 μg/ml uracil to select for pop-out clones. Knockout candidates were identified by PCR using the same primers as mentioned above and the additional primer pair Cas1 *Eco*RV fw/Cas1 *Apa*I rev. Homozygous deletion strains were finally confirmed via Southern Blot analysis (Sambrook and Russell, 2001; Klein, 2011).

In order to establish a Δ*cas1*Δ*fen1* knockout strain, the Δ*cas1* strain was transformed with *fen1* deletion plasmid pCN6 (Meslet-Cladiére et al., 2007). Transformants were selected by plating on Hv-Ca medium supplemented with tryptophan (40 μg/ml). Pop-in candidates were subsequently plated onto Hv-Ca agar plates supplemented with L-tryptophan (40 μg/ml), 5-FOA (10 μg/ml) and 10 μg/ml uracil. Pop-in and pop-out clones were identified by PCR using the primers Fen1 HVO UP/Fen1 HVO DO and Fen1 *Sna*BI fw/Fen1 *Xba*I rev. Finally, homozygous pop-out candidates were verified by Southern Blot analysis (Sambrook and Russell, 2001) (Supplementary Figure 3). Genomic DNAs of candidate clones K1, K4, K17, K20, K22 and of wild-type H119 were extracted via spooling method (Dyall-Smith, 2008). Concentrations of genomic DNAs were determined with a NanoPhotometer N60, the DNA was stored at 4°C. Genomic DNA was digested with *Sal*I and a Southern blot analysis was made as described in Sambrook and Russell (2001). Radioactively labeled PCR products were used as hybridisation probes to detect DNA fragments on the southern blot membrane. PCR products amplified with Fen1 Sonde fw/Fen1 Sonde rev and Fen1 (HVO) UP/Fen1 (HVO) DO, respectively, using H119 gDNA as a template were used as probes. For radiolabeling, 50 μCi [α-^32^P]-dCTP and the DECAprime™ II DNA labeling kit (Life Technologies/Merck, Germany) were used. Two separate probes were generated, one that binds upstream of the *fen1* coding sequence, and another one that binds directly in the *fen1* gene. After hybrididsation the membrane was exposed to an x-ray film (Supplementary Figure 3).

### H_2_O_2_ and UV Radiation Survival Assays

Wild-type and deletion strains were inoculated in 4 ml Hv-YPC medium, strains that carried a plasmid were inoculated in 4 ml selective medium with the appropriate supplements and incubated over night at 45°C until culture density reached an OD_650nm_ of 0.3–0.5. To determine survival rates after H_2_O_2_ exposure, cultures that reached the adequate OD_650nm_ were aliquoted into two 2 ml reaction tubes by pipetting 490 μl culture into each tube. One tube was supplemented with 10 μl of a 200 mM H_2_O_2_ stock solution (final concentration 4 mM), and 10 μl of 18% saltwater was added to the other tube (control). H_2_O_2_ stock solutions were freshly prepared for each experiment by diluting 30% H_2_O_2_ with 18% saltwater. Samples were incubated at 45°C and 450 rpm for 1h. Subsequently, samples were serially diluted (1:10) in 18% saltwater and 20 μl of the 10^–3^ to 10^–6^ dilutions were spotted onto prewarmed Hv-YPC agar plates in duplicate. After the liquid was absorbed by the agar, plates were incubated at 45°C for 3 days until colonies could be counted. Survival rates were determined by dividing the number of colonies of the 4 mM H_2_O_2_ approach by the number of colonies of the control. P-values were determined by *t*-tests (two sample assuming equal variances).

To compare the sensitivity of the wild-type and deletions strains toward UV radiation, strains were incubated as described above until an OD_650nm_ of 0.3–0.5. Next, cultures were serially diluted (1:10) in 18% saltwater and 20 μl of the dilutions 10^–2^ to 10^–6^ were spotted onto Hv-YPC agar plates in duplicate. After the liquid was absorbed by the agar, plates were exposed to UV radiation (either 30 or 60 J m^–2^) in a Stratalinker^®^ UV Crosslinker (Stratagene, Agilent, Santa Clara, CA, United States). Control plates were not exposed to UV radiation. To prevent photoreactivation, all plates were incubated in the dark at 45°C for at least three days. Colonies were counted and survival rates were calculated by dividing the number of colonies on irradiated plates by the number of colonies on the control plates. All experiments were performed in triplicate.

### Light Microscopy

Liquid cultures were inoculated in 4 ml Hv-YPC medium and incubated at 45°C overnight. After cultures reached an OD_650nm_ of 0.3–0.5 (exponential phase) and 0.9–1.2 (stationary phase), respectively, 1 ml of each culture was transferred into a reaction tube and centrifuged at 1,500 x *g* for 10 min. The supernatant was discarded, and the obtained pellets were resuspended in 50 μl (derived from exponential culture) and 150 μl (derived from stationary culture) Hv-YPC medium, respectively, and applied onto agarose-covered microscope slides. Agarose slides were prepared as follows: 0.1 g agarose was solved in 4 ml bidestilled water by heating the suspension in a microwave until the agarose was completely dissolved. Meanwhile, 6 ml 18% saltwater was prewarmed in a water bath and added to the hot agarose solution. With the help of a brush, the agarose solution was applied onto the slides. 2 μl of the resuspended pellets were pipetted onto the hardened agarose slides and covered with a coverslip. The edges of the cover glasses were sealed with clear nail polish and microscopic analysis was done at 100 x magnification with oil immersion on a Leica DM5500 B light microscope.

### Scanning Electron Microscopy

For scanning electron microscopy H119 and Δ*cas1*Δ*fen1* cells were grown to stationary phase (OD_650nm_: 1.2) in Hv-YPC, adsorbed on silicon platelets and fixed for 1 h with a final concentration of 2.5% glutaraldehyde in 0.1 M phosphate buffer with 1% saccharose. Afterward, samples were fixed with 2% of osmium tetroxide in phosphate buffer for 20 min, dehydrated in a graded series of propanol and then critical point dried using carbon dioxide in a CPD BalTec030 critical point dryer. Finally, samples were mounted on specimen stubs and rotary coated by electron beam evaporation with about 2 nm of platinum. Samples were imaged in a Hitachi S-5200 scanning electron microscope at an accelerating voltage of 10 kV using the secondary electron signal.

### Co-immunoprecipitation and Mass Spectrometry

The FLAG-Cas1 fusion protein and as a control the FLAG protein were expressed in *Haloferax* cells (strain Δ*cas1*). 500 ml Hv-Ca medium supplemented with 40 μg/ml L-tryptophan were inoculated from a plate and grown until OD_650nm_ of 0.7. After incubation for 1 h cultures were harvested by centrifugation at 10,000 x *g* for 30 min at 4°C. The resulting pellets were washed with enriched PBS [2.5 M NaCl, 150 mM MgCl_2_,1xPBS (137 mM NaCl, 2.7 mM KCl, 10 mM Na_2_PO_4_, 2 mM KHPO_4_, pH 7.4)] and stored at −80°C. Pellets were thawed on ice and resuspended in 10 ml lysis buffer (50 mM Tris-HCl, pH 7.4, 1 mM EDTA, 10 mM MgCl_2_,1 mM CaCl_2_) containing 150 μl of proteinase inhibitor (Sigma-Aldrich/Merck, Darmstadt, Germany) and 100 μl RQ1 RNase-free DNase (Promega, Walldorf, Germany). After 30 min incubation on ice, cells were disrupted by ultrasonic treatment and subsequently centrifuged at 100,000 x *g* at 4°C for 30 min. The supernatants (S100) were transferred into fresh falcons and RNase A was added at a final concentration of 400 μg/ml. After incubation for 30 min at 37°C, NaCl was added to a final concentration of 150 mM. A total of one FLAG control purification and two FLAG-Cas1 purifications were carried out.

For affinity purification, 400 μl of anti-FLAG M2 affinity gel (Sigma-Aldrich/Merck, Darmstadt, Germany) was washed 10 times with 10 ml of ice-cold washing buffer (50 mM Tris/HCl, pH 7.4, 150 mM NaCl) before the lysate was added. After incubation overnight at 4°C, anti-FLAG M2 affinity gel was washed 10 times with 10 ml of ice-cold washing buffer. Proteins were eluted from the affinity gel using washing buffer supplemented with 150 ng/l 3xFLAG peptide and fractions were collected in 2 ml reaction tubes and finally precipitated using acetone (99%). Protein concentrations were determined using ROTI^®^Nanoquant according to the manufacturer’s protocol, extracts were snap frozen in liquid nitrogen and stored at −80°C. The FLAG elution fraction was concentrated using an Amicon Ultra – 4 10,000 NMWL device (centrifugation for 15 min at 7,500 x*g* and 4°C). 50 mM Na_3_PO_4_/150 mM NaCl buffer was added to the retentate to a final volume of 200 μl. The sample was then loaded onto a SuperdexTM 75 Increase 10/300 gel filtration column. 1 ml fractions containing Cas1 and eluting with the complex size of approximately 670 kDa were collected and used for mass spectrometry analyses.

SDS-PAGE-separated protein samples were processed as described by Shevchenko et al. (1996). The resulting peptides were loaded to nano HPLC coupled with Exploris Orbitrap Mass spectrometer (Thermo Fisher Scientific, Waltham United States). The peptides were separated with a linear gradient of 5–40% buffer B (80% acetonitrile and 0.1% formic acid) at flow rate of 300 nL/min over 48 min total gradient time. The MS data was aquired by scanning the precursors in mass range from 350 to 1400 m/z at a resolution of 60,000 at m/z 200. Top30 precursor ion were chosen for MS2 by using data-dependent acquisition (DDA) mode at a resolution of 15,000 at m/z 200 with auto IT. Data analysis and search was performed against Uniprot_Haloferax_volcanii database (July 2021, 3911 entries) using Maxquant Software (1.6.17.0) and results were annotated with Scaffold5 software. Search parameters were 0.1 FDR on peptide and protein level; Carbamidomethyl was set as fixed modification and oxidation of methionine and acetylation at the N-terminus of the protein as variable modifications.

To evaluate the data, first all proteins for which more than five peptides were found in the FLAG control were removed from the final tables (Table 3 and Supplementary Table 3). In addition, for proteins that were found in the FLAG only control (identified with four or less peptides) the number of peptides found in the FLAG only control was subtracted from those found in the FLAG-Cas1 samples. In addition, only proteins found in both FLAG-Cas1 purifications were included in Table 3 and Supplementary Table 3.

### Flow Cytometry

DNA content and cell size of *H. volcanii* cells were determined via flow cytometry. Cultures were prepared in 5 ml Hv-YPC broth and grown at 45°C with 8 rpm rotation in two successive overnight dilutions until an OD_650nm_ of ∼0.4 was reached. Acridine orange solution was added to the cells at a final concentration of 1 μg/ml. Samples were analysed using an FC500 flow cytometer (Beckman Coulter; University of Nottingham Flow Cytometry facility) equipped with a 488 nm laser and 528/28 emission filter to measure acridine orange fluorescence. Samples were run on the lowest speed setting and at least 20,000 cells were acquired for each sample. Data was analysed using Flow Jo v7.6 (Tree Star Inc.). Cells were gated based on forward and side scatter and doublets were excluded by height/area analysis.

### Preparation of Cell Extracts and Nuclease Assays

For the preparation of total protein extracts, 150 ml cultures were grown in Hv-YPC medium (strains without plasmids) or Hv-Min medium (strains carrying a plasmid) until they reached an OD_650nm_ of 0.6–0.8. Cells were harvested by centrifugation at 10.000 x *g* and 4°C for 30 min, cell pellets were washed once with enriched PBS [2.5 M NaCl, 150 mM MgCl_2_,1xPBS (137 mM NaCl, 2.7 mM KCl, 10 mM Na_2_PO_4_, 2 mM KHPO_4_, pH 7.4)] and stored at −80°C until further usage. Pellets were thawed on ice and resuspended in 2 ml Lysis buffer (100 mM Tris-HCl pH 7.5, 10 mM EDTA) and subsequently disrupted by ultrasonic treatment. To separate soluble and non-soluble lysate fractions, suspensions were centrifuged at 100,000 x *g* and 4°C for 30 min. The supernatants (S100) were transferred into 2 ml reaction tubes and total protein concentration was measured using ROTI^®^ Nanoquant solution according to the manufacturer’s instructions. Extracts were snap frozen in liquid nitrogen and stored at −80°C until further use.

For the *in vitro* 5′ flap processing experiments, 20 μg of the S100 protein extracts were mixed with 150 ng double flap DNA substrate and 10x IVP buffer (500 Tris-HCl pH 7.4, 500 mM MgCl_2_, 50 mM KCl) and incubated for 2 h at 45°C. The longer flap strand was ordered as oligonucleotide labelled with Cy5 at the 3′ end (Sigma-Aldrich, Taufkirchen, Germany). (Figure 5, as described in Craggs et al., 2014). Proteins were inactivated and DNA was denatured by heat treatment (90°C, 10 min) and reactions were loaded onto a denaturing 10% PAA gel. Cy5 signals were detected on a ChemiDoc using standard parameters for Cy5.

### RNA Sequencing

Three replicates of wild-type H119 and deletion strain Δ*cas1*Δ*fen1* were cultured in Hv-YPC medium and grown to OD_650nm_ of 0.6–0.7. Total RNA was isolated using NucleoZOL™ (Machery and Nagel, Düren, Germany) and RNA samples were sent to vertis Biotechnologie AG (Martinsried, Germany) for sequencing. Total RNA was treated with T4 Polynucleotide Kinase (NEB, Frankfurt, Germany) and rRNA was depleted using an in-house protocol. Samples were fragmented using an ultrasonic treatment before cDNA library was prepared. For this, first-strand cDNA synthesis was performed using a 3′-adapter primer and the M-MLV reverse transcriptase. After cDNA purification, the 5′ Illumina TruSeq sequencing adapter was ligated to the 3′ end of the antisense cDNA and the sample amplified using TruSeq_Sense_primer and TruSeq_Antisense_primer to 10–20 ng/μl using a high-fidelity DNA polymerase. Finally, cDNA was purified using the Agencourt AMPure XP kit (Beckman Coulter Genomics), samples were pooled (equimolar) and the pool size was fractionated (200–550 bp) by preparative agarose gel electrophoresis before the samples were sequenced on an Illumina NextSeq 500 system using 1 × 75 bp read length.

### Bioinformatics Analyses

Quality control was conducted on raw reads and after each processing step using fastqc (v0.11.9) (^2^accessed 09/09/20) and MultiQC (v1.10.1) (Ewels et al., 2016). Adapters were trimmed using trimgalore (v0.6.3) (^3^accessed 09/09/20) and reads were mapped against the *H. volcanii* genome with STAR (v2.7.3a) (Dobin et al., 2013). Unique reads were extracted and counted using featureCounts (v2.0.1) (Liao et al., 2014) and DESeq2 (v1.30.0) (Love et al., 2014) was used for differential expression analysis. Command lines for the processing steps are available in the Supplementary Material. A modular workflow assembler which was used to generate workflows for this analysis is available at https://github.com/jfallmann/MONSDA (Fallmann et al., 2022).

## Data Availability Statement

The datasets presented in this study can be found in online repositories. The names of the repository/repositories and accession number(s) can be found below: https://www.ncbi.nlm.nih.gov/, PRJNA776127; https://www.ebi.ac.uk/pride/archive/, PXD029952 (Vizcaíno et al., 2016).

## Author Contributions

JW, VS, SK, and TT did the experiments. JF, SK, PW, HU, PS, TA, FH, and AM performed data curation. AM conceptualised the project. FH and AM wrote the original draft. FH, JW, JF, SK, VS, TT, PW, HU, PS, TA, and AM reviewed and edited the draft. PW, HU, PS, TA, and AM provided the resources and funding. All authors contributed to the article and approved the submitted version.

## Conflict of Interest

The authors declare that the research was conducted in the absence of any commercial or financial relationships that could be construed as a potential conflict of interest.

## Publisher’s Note

All claims expressed in this article are solely those of the authors and do not necessarily represent those of their affiliated organizations, or those of the publisher, the editors and the reviewers. Any product that may be evaluated in this article, or claim that may be made by its manufacturer, is not guaranteed or endorsed by the publisher.

## Footnotes

1 Standard growth conditions (18% salt water) correspond to 15% NaCl, low salt (15% salt water) corresponds to 10.8% NaCl and high salt (23% salt water) corresponds to 19.2% NaCl.

2
https://www.bioinformatics.babraham.ac.uk/projects/fastqc/

3
https://github.com/FelixKrueger/TrimGalore
