# Supplementary material for: Cas1 and Fen1 Display Equivalent Functions During Archaeal DNA Repair
Source: Front Microbiol. 2022 Apr 15;13:822304. doi: 10.3389/fmicb.2022.822304 (PMC9051519; doi:10.3389/fmicb.2022.822304)
Supplement: Supplementary file 1 [file Data_Sheet_1.pdf]

Supplementary Data

Page

Supplementary Methods 2

Supplementary Tables 3

Supplementary Table 1. Up-regulated genes in *Δcas1Δfen1* 3
Supplementary Table 2. Down-regulated genes in *Δcas1Δfen1* 6
Supplementary Table 3. Proteins copurifying with FLAG-Cas1 9
Supplementary Table 4. Strains used 10
Supplementary Table 5. Plasmids used 10
Supplementary Table 6. Oligonucleotides used 12

Supplementary Figures 14
Supplementary Figure 1 14
Supplementary Figure 2 15
Supplementary Figure 3 16

References 17

Supplementary Methods

CLI commands for RNA-seq read analysis

Trimming:

```
$trim_galore  --cores 4 --gzip -q 15 --length  20 -e 0.15 *.fastq.gz$
```

Create index:

```
$STAR  --runMode  genomeGenerate  --runThreadN  16 --
genomeDirGENOMES/HaVo/star  --genomeFastaFiles  GENOMES/HaVo/havo.fa --
sjdbGTFfile  GENOMES/HaVo/havo.gtf  --genomeSAindexNbases 5 --
sjdbGTFfeatureExon  CDS  --sjdbGTFtagExonParentTranscript  Parent$
```

Mapping:

```
$STAR  --outSAMprimaryFlag  AllBestScore  --runThreadN  14 --
outSAMattributes  NH HI AS nM MD --genomeDir  GENOMES/HaVo/star--
readFilesCommand  zcat  --readFilesIn  FASTQ /*.fq.gz --outReadsUnmapped
Fastx  --limitOutSJcollapsed  300000000  --limitIObufferSize  8200000000  -
-outFilterMultimapNmax  30 --sjdbGTFfeatureExon  CDS  --
sjdbGTFtagExonParentTranscript  Parent$
```

Trimming:

```
$featureCounts  -T 16 -f --fraction  -O -M -a HaVo.gtf *.sam$
```

Supplementary Tables

Supplementary Table 1. RNA expression profile of the Δ*cas1*Δ*fen1* deletion mutant compared to the wildtype. Shown are all up-regulated genes.

gene ID	annotation/ gene name	logFC[1]	p_{adj}-value[2]	functional group[3]
HVO_2933	hypothetical protein	2,58	4,23E-08	S
HVO_3040	23S ribosomal RNA	2,39	2,13E-02	rRNA
HVO_3064	16S ribosomal RNA	2,32	2,38E-03	rRNA
HVO_B0028	xylose dehydrogenase (NAD/NADP), *xacA*	2,32	4,84E-04	S
HVO_B0231	hypothetical protein	2,23	4,56E-03	S
HVO_0515	hypothetical protein	2,23	1,52E-07	S
HVO_B0342	oxidoreductase (luciferase family protein), *mer2*	2,19	1,56E-03	C
HVO_0369	hypothetical protein; ProVir2 prediction	2,18	8,32E-03	S
HVO_1434	hypothetical protein; ProVir5 prediction	2,16	6,56E-11	S
HVO_0641	cob(II)yrinic acid a,c-diamide reductase, *bluB*	2,10	4,71E-07	C
HVO_B0343	hydrolytic enzyme LplD, *lplD*	2,07	2,85E-02	G
HVO_B0304	hypothetical protein	2,07	2,11E-02	S
HVO_2817	transposase (ISH51)	2,06	1,67E-08	L
HVO_A0258	ISH4-type transposase homolog	2,06	1,91E-04	L
HVO_0694	purine phosphoribosyl-transferase, *gptA*	2,05	5,48E-04	F
HVO_1721	2Fe-2S iron-sulfur cluster binding domain-containing protein, *ferA3*	2,04	1,08E-07	C
HVO_A0455	hypothetical protein	2,02	1,37E-05	S
HVO_A0218	oxidoreductase; ProVir4 prediction	2,01	1,51E-04	S
HVO_0276A	homolog to HGPV1-ORF9; ProVir6 prediction	2,00	5,46E-04	S
HVO_1687	hypothetical protein	2,00	1,15E-10	S
HVO_1205	taxis cluster protein CheD, *cheD*	2,00	2,23E-04	N
HVO_2935	hypothetical protein	1,98	4,06E-13	S
HVO_2011	hypothetical protein	1,96	5,12E-09	S
HVO_0279	hypothetical protein; ProVir6 prediction	1,95	5,46E-05	S
HVO_B0125	ABC-type transport system ATP-binding protein (probable substrate dipeptide /oligopeptide), *dppD13*	1,95	1,16E-02	E
HVO_2934	hypothetical protein	1,91	1,54E-07	S
HVO_2964	hypothetical protein	1,90	2,17E-06	S
HVO_A0026	transcription initiation factor TFB, ProVir3, *tfb9*	1,87	1,13E-05	K
HVO_0526	hypothetical protein	1,86	1,94E-05	S
HVO_B0280	hypothetical protein	1,85	1,29E-02	S
HVO_B0380	putative transcription activator, *tenA1*	1,85	2,10E-03	K
HVO_3038	16S ribosomal RNA	1,85	7,08E-03	rRNA

[1] logFC: log_2 fold change

[2] p_{adj}-value: adjusted p-value

[3] Letter code: C: energy production and conversion; E: amino acid transport and metabolism; F: nucleotide transport and metabolism; G: carbohydrate transport and metabolism; I: lipid transport and metabolism; J: translation, ribosomal structure and biogenesis; K: transcription; L: replication, recombination and repair; O: post-translational modification, protein turnover, chaperons; P: inorganic ion transport and metabolism; Q: secondary metabolites biosynthesis, transport and catabolism; S: function unknown; T: signal transduction; U: intracellular trafficking, secretion and vesicular transport

gene ID	annotation/ gene name	logFC[1]	p_{adj}-value[2]	functional group[3]
HVO_A0098	transcription initiation factor TFB, *tfb12*	1,82	8,99E-03	K
HVO_2628	beta-ribofuranosylamino-benzene 5'-phosphate synthase, *pabA*	1,81	3,71E-04	P
HVO_0153	urease accessory protein UreF, *ureF*	1,80	1,00E-03	O
HVO_C0069	GFO family oxidoreductase, *gfo1*	1,79	5,29E-06	L
HVO_0280	hypothetical protein; ProVir6 prediction	1,74	9,67E-06	S
HVO_B0091	ABC-type transport system permease protein(Probable substrate dipeptide/ oligopeptide), *dppC12*	1,74	4,18E-03	P
HVO_1434A	hypothetical protein	1,73	2,97E-09	S
HVO_2489	small CPxCG-related zinc finger protein	1,73	3,94E-04	S
HVO_B0201	ArcR family transcription regulator, *arcR18*	1,72	2,94E-04	K
HVO_C0062	hypothetical protein	1,72	1,26E-04	S
HVO_2295	homolog to cationic amino acid transport protein N-terminal region	1,71	3,91E-03	E
HVO_0363	hypothetical protein; ProVir2 prediction	1,71	1,73E-04	S
HVO_A0215	putative membrane-bound metal-dependent hydrolase	1,71	1,18E-07	S
HVO_1640	pseudogene	1,71	2,64E-08	-
HVO_0169	TrmB family transcription regulator	1,70	8,52E-08	K
HVO_0365	hypothetical protein; ProVir2 prediction	1,70	3,47E-04	S
HVO_2570	small CPxCG-related zinc finger protein	1,69	1,05E-02	S
HVO_0226	transcription initiation factor IIB, *tfb7*	1,68	2,86E-03	K
HVO_B0332	ABC-type transport system ATP-binding protein (probable substrate dipeptide/ oligopeptide), *dppF15*	1,68	1,80E-02	E
HVO_0605	ArcR family transcription regulator, *arcR1*	1,68	8,50E-04	K
HVO_C0030	hypothetical protein	1,67	1,40E-07	S
HVO_B0219	ABC-type transport system permease protein(Probable substrate branched-chain amino acids), *livM6*	1,67	5,27E-03	E
HVO_B0210	putative isomerase	1,67	1,46E-02	G
HVO_0267	hypothetical protein; ProVir6 prediction	1,67	2,34E-05	S
HVO_2772	rhodanese domain protein / beta-lactamase domainprotein	1,66	2,44E-04	P
HVO_A0408A	hypothetical protein	1,65	2,28E-03	S
HVO_A0251	hypothetical protein; ProVir4 prediction	1,65	2,00E-11	S
HVO_A0599	hypothetical protein	1,65	1,36E-02	S
HVO_0366	hypothetical protein; ProVir2 prediction	1,62	8,93E-04	S
HVO_B0305	hypothetical protein	1,61	2,14E-02	S
HVO_0341	hypothetical protein, predicted ATPasee	1,60	5,44E-05	S
HVO_A0490	family 3 CoA transferase	1,60	2,10E-05	C
HVO_B0319	ArcR family transcription regulator, *arcR20*	1,60	3,12E-03	K
HVO_0611	tricarboxylate transport membrane protein RctA	1,60	1,68E-03	S
HVO_0268	hypothetical protein; ProVir6 prediction	1,60	5,03E-03	S
HVO_A0325	cellulase	1,59	9,79E-04	C
HVO_0275	transposase (ISH5); ProVir6 prediction	1,59	7,27E-03	L
HVO_B0325	cellulase	1,58	9,77E-03	C
HVO_0502	hypothetical protein	1,58	8,95E-05	S
HVO_B0222	PQQ enzyme repeat domain protein	1,57	1,09E-02	S
HVO_A0634	M20 peptidase family protein	1,56	1,59E-03	E

gene ID	annotation/ gene name	logFC[1]	p_{adj}-value[2]	functional group[3]
HVO_C0005A	homolog to virus structural protein HGPV1-VP3 (nonfunctional)	1,55	1,23E-03	-
HVO_B0306	amidase (Hydantoinase/carbamoylase family), *amaB4*	1,55	6,50E-03	E
HVO_2106	fructose-bisphosphate aldolase	1,55	3,21E-03	G
HVO_B0089	ABC-type transport system ATP-binding protein (probable substrate dipeptide/ oligopeptide), *dppF12*	1,55	6,15E-03	E
HVO_2754	integral membrane protein superfamily	1,54	3,29E-04	S
HVO_0274	hypothetical protein; ProVir6 prediction	1,54	7,57E-04	S
HVO_3008	hypothetical protein, Zn-finger containing protein	1,54	3,49E-05	S
HVO_A0309	ISH7-type transposase HfIRS6 (non functional)	1,54	1,17E-02	-
HVO_2470	sodium- and chloride-dependent transporter, *nac*	1,54	4,86E-13	S
HVO_A0220	putative nucleotide sugar epimerase; ProVir4 prediction, *wecB*	1,53	1,65E-03	M
HVO_1627	hypothetical protein	1,53	1,74E-03	S
HVO_0881	signal peptide peptidase SppA, *sppA1*	1,53	5,39E-04	O
HVO_2717	ambigous_alaS2, *alaS2*	1,52	8,96E-08	J
HVO_1953	11-domain light and oxygen sensing his kinase	1,52	1,91E-04	T
HVO_1734A	Restriction endonuclease domain protein	1,52	1,18E-10	S
HVO_2461	CBS domain protein, *gul*	1,51	1,10E-04	S
HVO_B0119	ArcR family transcription regulator, *arcR17*	1,50	3,23E-03	K
HVO_A0395	hypothetical protein	1,50	3,86E-04	S

Supplementary Table 2. RNA expression profile of the Δ*cas1*Δ*fen1* deletion mutant compared to the wildtype. Shown are all down-regulated genes.

gene ID	annotation/ gene name	logFC[1]	p_{adj}-value[2]	functional group[3]
HVO_2873	flap endonuclease, *fen1*[4]	-8,13	2,59E-54	L
HVO_A0211	Cas1, *cas1*[3]	-7,63	2,05E-38	L
HVO_A0279	transposase (ISH18)	-4,41	4,04E-51	L
HVO_2507	Asn family transcription regulator, *trh7*	-3,17	1,40E-07	K
HVO_2522	Asn family transcriptional regulator, *trh8*	-2,97	1,41E-10	K
HVO_0752	hypothetical protein	-2,96	3,29E-07	S
HVO_1228	halocyanin domain protein, *hcpE*	-2,92	4,69E-11	C
HVO_2606	PQQ repeat-containing protein	-2,72	1,35E-19	S
HVO_0910	hypothetical protein	-2,72	7,20E-09	S
HVO_1092	ribonuclease P protein component 2, *rnp2*	-2,68	1,31E-13	J
HVO_1873	hypothetical protein	-2,64	1,80E-10	S
HVO_2508	carbamoyl-phosphate synthase small subunit, *carA*	-2,56	2,20E-05	F
HVO_B0045	diaminobutyrate decarboxylase, *bdb*	-2,47	1,28E-05	E
HVO_A0541	ABC-type transport system periplasmic substrate-binding protein (probable substrate iron-III)	-2,45	1,64E-08	E
HVO_B0044	iucA iron transport protein A, *iucA*	-2,43	4,20E-08	Q
HVO_0325	hypothetical protein	-2,35	3,03E-08	S
HVO_3052	$tRNA^{Gly}$	-2,35	6,92E-08	tRNA
HVO_0799	hypothetical protein	-2,30	8,93E-08	S
HVO_2607	PQQ repeat-containing protein	-2,29	2,84E-17	S
HVO_2588	isocitrate dehydrogenase, *icD*	-2,28	2,02E-06	C
HVO_B0046	diaminobutyrate pyruvate amino-transferase, *dat*	-2,26	1,76E-11	E
HVO_2051	transposase (ISH51)	-2,23	5,74E-06	L
HVO_0537	hypothetical protein	-2,19	7,05E-11	S
HVO_2656	hypothetical protein	-2,19	1,22E-17	S
HVO_2354	hypothetical protein	-2,19	9,82E-05	S
HVO_1362	hypothetical protein	-2,07	1,05E-04	S
HVO_2361	carbamoyl-phosphate synthase large subunit, *carB*	-2,04	1,26E-04	F
HVO_2284	hypothetical protein	-1,97	2,59E-06	S
HVO_0050	hypothetical protein	-1,96	1,37E-12	S
HVO_1976	preprotein translocase subunit SecD, *secD*	-1,94	1,10E-06	U

[4] These genes were deleted in Δ*cas1*Δ*fen1*.

gene ID	annotation/ gene name	logFC[1]	p_{adj}-value[2]	functional group[3]
HVO_1701	hypothetical protein	-1,93	2,93E-08	S
HVO_2139	hypothetical protein	-1,93	2,72E-09	S
HVO_1045	hypothetical protein	-1,90	4,69E-13	S
HVO_2821	transposase (ISH18)	-1,90	4,39E-07	L
HVO_1863	hypothetical protein	-1,88	3,81E-19	S
HVO_1359	hypothetical protein	-1,87	2,80E-11	S
HVO_1606	hypothetical protein	-1,86	7,66E-09	S
HVO_B0198	ABC-type transport system periplasmic substrate-binding protein (probable substrate iron-III)	-1,83	1,03E-06	E
HVO_B0356	hypothetical protein	-1,82	2,19E-10	S
HVO_1229	hypothetical protein	-1,82	8,35E-07	S
HVO_2511	glutamyl-tRNA(Gln) amidotransferase subunit D, *gatD*	-1,81	4,98E-05	J
HVO_A0148	ABC-type transport system periplasmicsubstrate-binding protein (probable substrate sugar), *tsgA4*	-1,80	2,22E-03	G
HVO_1079	sulfate adenylyltransferase small subunit, *cysD1*	-1,77	9,38E-11	E
HVO_2655	Rieske iron-sulfur protein, *narB2*	-1,77	2,61E-10	C
HVO_3033	$tRNA^{Arg}$	-1,75	1,39E-08	tRNA
HVO_3016	$tRNA^{Arg}$	-1,74	4,08E-09	tRNA
HVO_A0542	hypothetical protein	-1,74	2,19E-04	S
HVO_2884	hypothetical protein	-1,72	2,60E-10	S
HVO_1758	thioredoxin reductase, *trxB5*	-1,72	8,10E-08	O
HVO_1162	sec-independent protein translocase protein TatAt, *tatAt*	-1,72	1,44E-05	U
HVO_A0151	transposase (ISH51)	-1,72	8,78E-05	L
HVO_1796	hypothetical protein	-1,70	3,46E-06	S
HVO_A0167	small CPxCG-related zinc finger protein	-1,67	3,84E-03	S
HVO_1186	hypothetical protein	-1,66	9,45E-10	S
HVO_0172	hypothetical protein	-1,65	1,85E-05	S
HVO_0660	hypothetical protein	-1,65	2,06E-05	S
HVO_0036	hypothetical protein	-1,65	1,85E-16	S
HVO_3027	$tRNA^{Tyr}$	-1,64	5,01E-06	tRNA
HVO_2443	putative dipeptides/oligopeptides ABC transporter ATP-binding protein, *dppD5*	-1,64	2,31E-05	E
HVO_1361	hypothetical protein	-1,63	2,99E-08	S
HVO_2037	hypothetical protein	-1,62	1,66E-07	S
HVO_1848A	small CPxCG-related zinc finger protein	-1,62	3,10E-09	S
HVO_1293	Tat (twin-arginine translocation) pathway signalsequence	-1,62	5,52E-07	S

gene ID	annotation/ gene name	logFC[1]	p_{adj}-value[2]	functional group[3]
HVO_B0358	hypothetical protein	-1,61	4,29E-06	S
HVO_2091	4-aminobutyrate aminotransferase, *gabT2*	-1,59	4,10E-06	E
HVO_A0551	acyl-CoA synthetase, *acs9*	-1,59	5,07E-06	I
HVO_A0558	ABC-type transport system periplasmic substrate-binding protein (probable substrate iron-III)	-1,58	5,22E-06	E
HVO_3029	$tRNA^{Met}$	-1,58	7,85E-07	tRNA
HVO_B0159	HTH-10 family transcription regulator NarO, *narO*	-1,58	4,59E-06	K
HVO_2838	receiver box response regulator	-1,57	1,26E-10	T
HVO_2751	hypothetical protein	-1,57	4,09E-07	S
HVO_0061	putative dipeptides/oligopeptides ABC transporter permease, *dppB1*	-1,56	1,78E-07	P
HVO_A0283	ABC-type transport system periplasmicsubstrate-binding protein (probable substrate sugar), *tsgA5*	-1,56	5,39E-07	G
HVO_0286	cinA N-terminal domain-containing protein	-1,56	9,22E-15	S
HVO_A0386	N-methylhydantoinase (ATP-hydrolyzing), *hyuB3*	-1,55	3,22E-09	E
HVO_2669	protoporphyrinogen oxidase, *hemG*	-1,53	3,75E-06	C
HVO_1647	cytochrome C oxidase subunit I	-1,51	2,48E-04	S
HVO_2749	50S ribosomal protein L21e, *rpl21e*	-1,51	4,93E-05	J
HVO_B0043	iron transport protein B, *iucB*	-1,51	6,64E-04	J
HVO_2892A	NADPH-dependent FMN reductase, *msuE*	-1,51	2,79E-05	S
HVO_1764	hypothetical protein	-1,51	2,23E-06	S
HVO_A0133	Tat (twin-arginine translocation) pathway signalsequence domain protein	-1,50	9,34E-09	S

Supplementary Table 3. Proteins copurified with FLAG-Cas1. Shown are proteins with their peptides sequenced by LC-MS and having unique spectra/peptide counts >30. Column "name" lists the gene name and column "peptides" shows the number of identified peptides. The complete list of identified proteins with their sequenced peptides has been uploaded to the PRIDE database with the dataset identifier PXD029952.

gene ID	name	annotation	peptides/ unique spectra counts
**replication and repair**			
HVO_0014	*hel308a*	ATP-dependent DNA helicase Hel308a	44
HVO_0854	*rad50*	DNA double-strand break repair ATPase Rad50	56
HVO_B0118	*sph2*	Smc-like protein Sph2; homologue of Rad50	30
HVO_0552	*muts1b*	DNA mismatch repair protein MutS	49
HVO_0393	*uvrA*	UvrABC system protein A	73
HVO_0029	*uvrB*	UvrABC system protein B	32
HVO_0415	*uvrD*	repair helicase UvrD	38
HVO_1018	*recJ3*	Hef-associated 3 exonuclease Han recJ3	35
HVO_2889	*recJ4*	DHH/RecJ family phosphoesterase RecJ4	33
HVO_2380	*cdc48a*	AAA-type ATPase (CDC48 subfamily)	66
HVO_0220	*mcm*	ATP-dependent DNA helicase MCM	34
HVO_0349	*rpoA1*	DNA-directed RNA polymerase subunit A	56
HVO_0347	*rpoB2*	DNA-directed RNA polymerase subunit B	44
HVO_0858	*polB1*	DNA-directed DNA polymerase B (intein-containing)	36
**Cas protein**			
HVO_A0206	*cas8*	CRISPR-associated protein Cas8b	58
**RNases**			
HVO_0874	*cpsf1*	zinc-dependent nuclease CPSF1	44
HVO_2724	*rnJ*	ribonuclease J	35
**sensing kinases**			
HVO_1811		sensor box histidine kinase	32
HVO_B0154		receiver/sensor box histidine kinase	41
**aminoacid synthesis and translation**			
HVO_0206	*alaS1*	alanine-tRNA synthetase	53
HVO_1547	*ileS*	isoleucine-tRNA synthetase	32
HVO_1684	*thrS*	threonine-tRNA synthetase	34
HVO_2361	*carB*	carbamoyl-phosphate synthase (glutamine-hydrolyzing) large subunit	43
HVO_2624	*pyrG*	CTP synthase	30
HVO_0869	*gltB*	glutamate synthase (ferredoxin) large subunit	89
HVO_2888	*elp3*	homolog to elongator complex protein ELP3	32
**vitamin metabolism**			
HVO_2452	*nrdJ*	Vitamin B12-dependent ribonucleotide reductase	68
HVO_B0050	*cobN*	ATP-dependent cobaltochelatase subunit CobN	73
HVO_B0051	*chlID*	ATP-dependent cobaltochelatase subunit ChlID	30
HVO_1088	*folCP*	folylpolyglutamate synthase / 7,8-dihydropteroate reductase / dihydropteroate synthase	38
**glucose metabolism**			
HVO_1955	*citB1*	aconitate hydratase	35
HVO_0541	*citB2*	aconitate hydratase	40
HVO_0812	*ppsA*	phosphoenolpyruvate synthase	53
HVO_2621	*ppc*	phosphoenolpyruvate carboxylase	66

gene ID	name	annotation	peptides/ unique spectra counts
**other**			
HVO_0316	*atpA*	A-type ATP synthase subunit A	33
HVO_0421		arNOG04375 family protein (homolog to PilT-type ATPase)	45
HVO_1735		SWIM zinc finger domain protein	35
HVO_2269	*rmeR*	type I site-specific deoxyribonuclease subunit RmeR	36
HVO_B0041	*iucC*	siderophore biosynthesis protein IucC	49

Supplementary Table 4. Strains used in this study.

strains	genotype	source/ reference
E. coli		
DH5α	F- φ80*lac*ZΔM15 Δ(*lac*ZYA-*arg*F) U169 *rec*A1 *end*A1 *hsd*R17 (rk-, mk+) *gal*- *pho*A *sup*E44 λ- *thi*-1 *gyr*A96 *rel*A1	Invitrogen (Thermo Fischer Scientific, Waltham, MA, USA)
H. volcanii		
H119	ΔpHV2Δ*pyrE2*Δ*leuB*Δ*trpA*	(Allers et al. 2004)
H26	ΔpHV2Δ*pyrE2*	(Allers et al. 2004)
Δ*fen1* (H938)	ΔpHV2Δ*pyrE2*Δ*fen1*	(Lestini, Duan, and Allers 2010)
Δ*cas1* (HV88)	ΔpHV2Δ*pyrE2*Δ*leuB*Δ*trpA*Δ*cas1*	(Klein 2011)
Δ*cas1*Δ*fen1* (HV95)	ΔpHV2Δ*pyrE2*Δ*leuB*Δ*trpA*Δ*cas1*Δ*fen1*	this study

Supplementary Table 5. Plasmids.

plasmid	relevant properties	source/ reference
**deletion strain generation**		
pCN6	*fen1* deletion plasmid, *fen1* deletion plasmid derived from pTA131 with up- and downstream regions of *fen1* (HVO_2873) coding sequence; ColE1 ori, f1 ori, Amp^R, *lacZ, pyrE2*	(Meslet-Cladiére et al. 2007)
pTA131-UP-Cas1-DO	plasmid containing *cas1* coding sequence (HVO_A0211) as well as 480 bp of the upstream and 587 bp of the downstream regions of *cas1;* ColE1 ori, f1 ori, Amp^R, *lacZ, pyrE2*	this study
pTA131-UPDO (*cas1*)	*cas1* deletion plasmid with 480 bp of the up- and 587 bp of the downstream regions of *cas1*	this study

plasmid	relevant properties	source/ reference
	(HVO_A0211) coding sequence; ColE1 ori, f1 ori, Amp^R, *lacZ, pyrE2*	
**cloning**		
pBlueskript II KS	ColE1 ori, f1 ori, *lacZ*, Amp^R	Stratagene
pBlue-NdeI-Cas1-EcoRV	ColE1 ori, f1 ori, *lacZ*, Amp^R; *cas1* gene (without TGA) insert with *Nde*I and *Eco*RV restriction sites	(Dierolf 2010)
pBlue-EcoRV-Cas1-XbaI	ColE1 ori, f1 ori, *lacZ*, Amp^R; *cas1* gene (without TGA) insert with *Ec*oRV and *Xba*I restriction sites	this study
pBlue-*Sna*BI-Fen1-XbaI	ColE1 ori, f1 ori, *lacZ*, Amp^R; *fen1* gene (without TGA) insert with *Sna*BI and *Xba*I restriction sites	this study
pMA-T-FdxSyn	ColE1 ori, f1 ori, Amp^R, p.fdx-promoter, t.syn terminator	GeneArt®
pTA131	ColE1 ori, f1 ori, Amp^R, *lacZ, pyrE2*	(Allers et al. 2004)
pTA927-NZ-NFLAG	ColE1 ori, f1 ori, Amp^R, pHV2 ori, *pyrE2*, p.tnaA-promoter, t.syn-terminator, ATG- 3x FLAG tag and gene HVO_2763 from *H. volcanii*	(Fischer 2009)
pTA231	ColE1 ori, f1 ori, Amp^R, pHV2 ori, *trpA*	(Allers et al. 2004)
pTA231-p.fdx	ColE1 ori, f1 ori, Amp^R, pHV2 ori, *trpA*, p.fdx-promoter, t.syn-terminator	this study
pTA230	ColE1 ori, f1 ori, Amp^R, pHV2 ori, *pyrE2*	(Allers et al. 2004)
pTA230-p.fdx-NFLAG	shuttle plasmid; ColE1 ori, f1 ori, Amp^R, pHV2 ori, *pyrE2*, p.fdx-promoter, t.syn-terminator, ATG-3x FLAG	this study
pTA231-p.fdx-NFLAG	ColE1 ori, f1 ori, Amp^R, pHV2 ori, *trpA*, p.fdx-promoter, t.syn-terminator, ATG-3x FLAG	this study
pTA962	ColE1 ori, f1 ori, *lacZ,* Amp^R, *pyrE2, hdrB,* pHV2 ori, p.tnaA-promoter, t.syn-terminator	(Allers et al. 2010)
**over-expression plasmids used for co-immunoprecipitations**		
pTA927-FLAGcontrol	ColE1 ori, f1 ori, Amp^R, pHV2 ori, *pyrE2*, p.tnaA-promoter, ATG-3x FLAG tag-TGA, t.syn-terminator	this study
pTA927-Cas1-NFLAG	ColE1 ori, f1 ori, Amp^R, pHV2 ori, *pyrE2*, p.tnaA-promoter, t.syn-terminator, ATG-3x FLAG tag and *cas1* gene (HVO_A0211) from *H. volcanii*	(Dierolf 2010)
**over-expression/ complementation plasmids used for H_2O_2 and UV assays**		
pTA230-p.fdx-Fen1-NFLAG	ColE1 ori, f1 ori, Amp^R, pHV2 ori, *pyrE2*, p.fdx-promoter, t.syn-terminator, ATG-3x FLAG and *fen1* gene (HVO_2873) from *H. volcanii*	this study
pTA230-p.fdx-Cas1-NFLAG	ColE1 ori, f1 ori, Amp^R, pHV2 ori, *pyrE2*, p.fdx-promoter, t.syn-terminator, ATG-3x FLAG and *cas1* gene (HVO_A0211) from *H. volcanii*	this study
pTA230-p.fdx-Cas1E158A-NFLAG	plasmid; ColE1 ori, f1 ori, Amp^R, pHV2 ori, *pyrE2*, p.tnaA-promoter t.syn-terminator, ATG-3x FLAG and *cas1*E158A mutant gene	this study
pTA962-p.fdx-Fen1-NFLAG-Cas1	ColE1 ori, f1 ori, *lacZ,* Amp^R, *pyrE2, hdrB,* pHV2 ori, p.fdx- promoter, t.syn-terminator, ATG-3x FLAG and *fen1* gene (HVO_2873) as	this study

plasmid	relevant properties	source/ reference
	well as *cas1* gene (HVO_A0211) from *H. volcanii*	
**over-expression/ complementation plasmids used for *in vitro* processing assays**		
pTA927-Cas1-CFLAG	ColE1 ori, f1 ori, Amp^R, pHV2 ori, *pyrE2*, p.tnaA-promoter, t.syn-terminator, 3x FLAG tag-TGA and *cas1* gene (HVO_A0211) from *H. volcanii*	(Dierolf 2010)
pTA927-Cas1E158A-CFLAG	ColE1 ori, f1 ori, Amp^R, pHV2 ori, *pyrE2*, p.tnaA-promoter, t.syn-terminator, 3x FLAG tag-TGA and *cas1*E158A mutant gene	this study
pTA230-p.fdx-Fen1-NFLAG	ColE1 ori, f1 ori, Amp^R, pHV2 ori, *pyrE2*, p.fdx-promoter, t.syn-terminator, ATG-3x FLAG and *fen1* gene (HVO_2873) from *H. volcanii*	this study

Supplementary Table 6. Oligonucleotides used in this study.

oligonucleotides	sequence (5'-3')	purpose
231NFlag fw (*Nde*I)	TTATAATCATATGGACTACAAAGACCATG	cloning
231NFlag rev (*Sna*BI *Xba*I)	ATTATAATCTAGAACATGTTACGTATTTATCGTC GTCATCTTT	cloning
FLAG NdeI fw	TATTACATATGGACTACAAAGACCATGAC	cloning
FLAG EcoRI rev	TATTAGAATTCATTTATCGTCGTCATC	cloning
Fen1 *SnaB*I fw	TATTAATACGTAGGAAACGCAGACCTGC	cloning and strain generation
Fen1 *Xba*I rev	ATAATATCTAGATACGTCCAGCGGTC	cloning and strain generation
Cas1 *EcoR*V fw	TATTAAGATATCACAAAAGCAAATCACCATATC	cloning
Cas1 *Xba*I rev	ATATTATCTAGATCACCACCACTTCTCCGT	cloning
Cas1up-FlagC (*Nde*I)	CCCTAATATATCATATGACAAAAGCAAATCACC ATATC	cloning
Cas1do-FlagC (*BamH*I)	TAATATGATATCCCACCACTTCTCCGTCGCG	cloning
Cas1E158A fw	GCTTCGAGCGGTCGCCGGAGACGCGAG	cloning
Cas1E158A rev	CTCGCGTCTCCGGCGACCGCTCGAAGC	cloning
5'ApaI p.fdx fw	TATTAGGGCCCTTCGTGGCAGTACGCTGGCCC	cloning
3'XmaJI Fen1+Stopp rev	TATTACCTAGGCTACGTCCAGCGGTCGA	cloning
5'XmaJI Cas1+Start fw	TATTACCTAGGATGACAAAAGCAAATCACC	cloning
3´BglIII Cas1+Stopp rev	TATTAAGATCTTCACCACCACTTCTCCGTCGC	cloning
Cas1KOUP	ATTACGGGCCTCATGGTGCAGTACTACC	strain generation
Cas1KODO	GTTGTTCCGTCTCGGCTTCCATCGACTCCGCT C	strain generation
iCas1 fw	[P]-ATGCAAGTTATCGTCGTCTACG	strain generation
iCas1 rev	[P]-gggTCACATCCAACAGAGGTCTTGGT	strain generation
Fen1 HVO UP	GCAGTTCGGCCATGGAGTTCGACC	strain generation, southern blot probe

Fen1 HVO DO	CAGCATTCCGTAGGTTGCCGAGAAG	strain generation, southern blot probe
Fen1 Sonde fw	GGACGAGTTCGGAGACGTACACG	southern blot probe
Fen1 Sonde rev	GAGAGGAGTTCGCGGCTGGTG	southern blot probe
Fen1 Substrat1 Cy5	TTTTTTTTATCTGACTGCCGTCTAGCTACTG-[Cy5]	ivp substrate
Fen1 Substrat2	CCTAGGTCCGTCCTAGCAAGCC	ivp substrate
Fen1 Substrat3	CAGTAGCTAGACGGCAGTCAGAGCTTGCTAGGACGGACCTAGGT	ivp substrate
Fen1 Substrat1 kurz Cy5	TCTGACTGCCGTCTAGCTACTG-[Cy5]	ivp size standard
TruSeq_Sense_primer	AATGATACGGCGACCACCGAGATCTACAC-NNNNNNNN-ACACTCTTTCCCTACACGACGCTCTTCCGATCT; NNN=i5 Barcode	RNA Seq
TruSeq_Antisense_primer	CAAGCAGAAGACGGCATACGAGAT-NNNNNNNN-GTGACTGGAGTTCAGACGTGTGCTCTTCCGATCT; NNN=i7 Index	RNA Seq
H119_1	i5 Barcode TCAGAGCC<br>i7 Barcode ATTACTCG	RNA Seq
H119_2	i5 Barcode CTTCGCCT<br>i7 Barcode ATTACTCG	RNA Seq
H119_3	i5 Barcode TAAGATTA<br>i7 Barcode ATTACTCG	RNA Seq
Δcas1Δfen1_1	i5 Barcode CTCCTTAC<br>i7 Barcode ATTCAGAA	RNA Seq
Δcas1Δfen1_2	i5 Barcode TATGCAGT<br>i7 Barcode ATTCAGAA	RNA Seq
Δcas1Δfen1_3	i5 Barcode TACTCCTT<br>i7 Barcode ATTCAGAA	RNA Seq

```
EcCas1      MTWLPLNPIPLKDRVSMIFLQYGQIDVIDGAFVLIDKTGI-RTHIPVGSVACIMLEPGTR        59
HvCas1      -----------MTKANHHIFADGELSRKEGTLRIDTLEGE-TKYLPVESIDALYLHGQIS        48
SsCas1      -----------MDKKIAFVKDYGAYLKVEKGLITCKIKNQVKWSIAPTELHSIVVLTNSS        49
AfCas1      --------------MRLVVDGFGKYLGIENGLIVVKEKGKALRKVRPEDLKQVLIIGKAA        46
                              .   *     :  :      .     :   .:  : :

EcCas1      VSHAAVRLAAQVGTLLVWVGEAGVR---VYASGQPGGARSDKLLYQAKLALDEDL-----        111
HvCas1      FNTRALGLLNKHGVPVHIFGWKDYYRGSYLPKRSQLSG---NTVVEQVRAYDNTERRLRI        105
SsCas1      ISSEVVKVANEYGIEIVFFNKHEPYAKLIP-AKYAGSF---KVWLKQLTAW--KRRKVEF        103
AfCas1      ISSDAIKLLLKNRVDVVFLDFNGEILGRLS-HPLIGTA---KTRREQYLAYG-DKRGVHL        101
            ..  .: :  :    :  ..                     :   :   *

EcCas1      -----RLKVVRKM-----FELRFGE----------------------------PAPARRS        133
HvCas1      GHRIIEASIHNMRANLQYYSGRRGDFDS---------------VVETLRELKTAVSDTQR        150
SsCas1      AKAFIYGKVHNQWVTLRYYERKY----------------GYNLTSQELDRLAREITFVNT        147
AfCas1      AKEFIKAKMANQMAILTNLAKARKDSNPEVAESLLKAKKEIDACLNELDGVEAE-MIDKV        160
                   .: .                                               .

EcCas1      VEQLRGIEGSRVRATYALLAK--QYGVTWNGRRYDPKD--WEKGDTINQCISAATSCLYG        189
HvCas1      IDELRAVEGDARKRYYDCFDSILEAPFRLAKREYNP---P---SNETNALISFLNGMVYT        204
SsCas1      AEEVMQKEAEAAKVYWRGVKSLLPKSLGFKGRMKRVS----DNLDPFNRALNIGYGMLRK        203
AfCas1      RERLLGIEGKASKHYWDAISLVIPEEYRFNGRRGIEIGSPRYAKDIVNAMLNYGYSILLA        220
             :.:   *..  :  :  .            *            :  *  :.   . :

EcCas1      VTEAAILAAGYAPAIGFVHTGKP--LSFVYDIADIIKFDTVVPKAFEIARRNPGEPDREV        247
HvCas1      SCVSAIRKTALDPTVGFVHEPGERRFTLSLDIADIFKPILADRLVFRLVNRKQITTDDFE        264
SsCas1      VVWGAVISVGLNPYIGFLHKFRSGRISLVFDLMEEFRSPFVDRKLIGLARESADKVTDLK        263
AfCas1      ECVKAVELAGLDPYAGFLHVDVSGRSSLAIDLMENFRQQVVDRVVLRLISYRQIKPEDCE        280
                *:  ..  *  **:*       ::  *: : ::   .    : :

EcCas1      RLACRDIFRSSKTLAKLIPLIEDVLAAGEIQPPAPPEDAQPVAIPLPV----------SL        297
HvCas1      TELAGCLLTEQGRLTVLEEFER-SLDQTV-QHPR---LKRKVSFKTLIQTDVYSLKKHLL        319
SsCas1      T--VYSLFSD--------------VKEDE------------------IYTQARRLVNAIL        289
AfCas1      KRNMVCQLSDNARRLLLASLLE-RLDSKT-QY-----RGRNLAYSSIILLHARDVVAFLR        333
                   : .              :                      :

EcCas1      GDAGHRSS----         305
HvCas1      TGEPYHATEKWW         331
SsCas1      NDEEYRPYLAK-         300
AfCas1      GERRYEGFVQKW         345
                :.
```

Supplementary Figure 1. Alignment of a bacterial and three archaeal Cas protein sequences. Cas protein sequences from *E. coli* (EcCas1)(Babu et al. 2011), *H. volcanii* (HvCas1, HVO_A0211), *Sulfolobus solfataricus* (Sscas1)(Rollie et al. 2015) and *Archaeoglobus fulgidus* (AfCas1)(Kim et al. 2013) were aligned using Clustal Omega (Goujon et al. 2010; McWilliam et al. 2013; Sievers et al. 2011). One of the conserved amino acids is E158 (HvCas1), which has been shown to be involved in the enzymatic activity (Babu et al. 2011; Kim et al. 2013; Rollie et al. 2015). Amino acids marked with an asterisk: positions with a single, fully conserved residue; marked with a colon: positions with conservation between amino acid groups of similar properties and marked with a period: positions with conservation between amino acid groups of weakly similar properties.

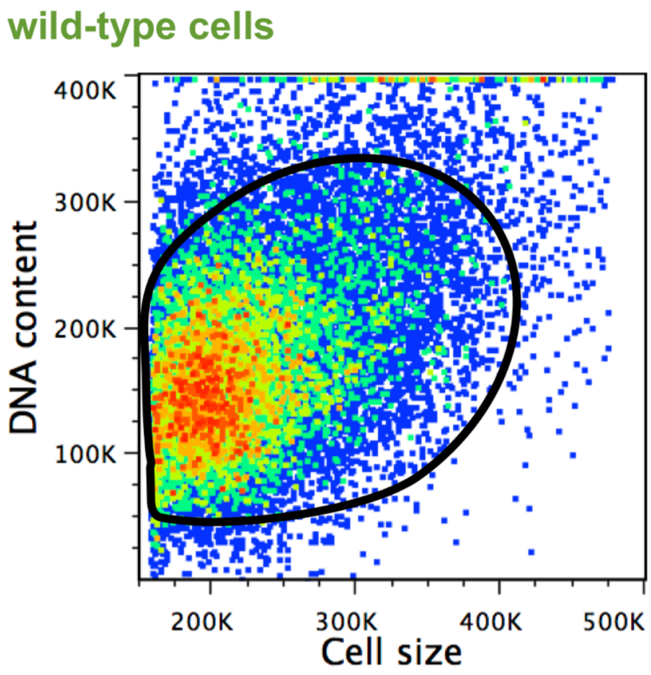

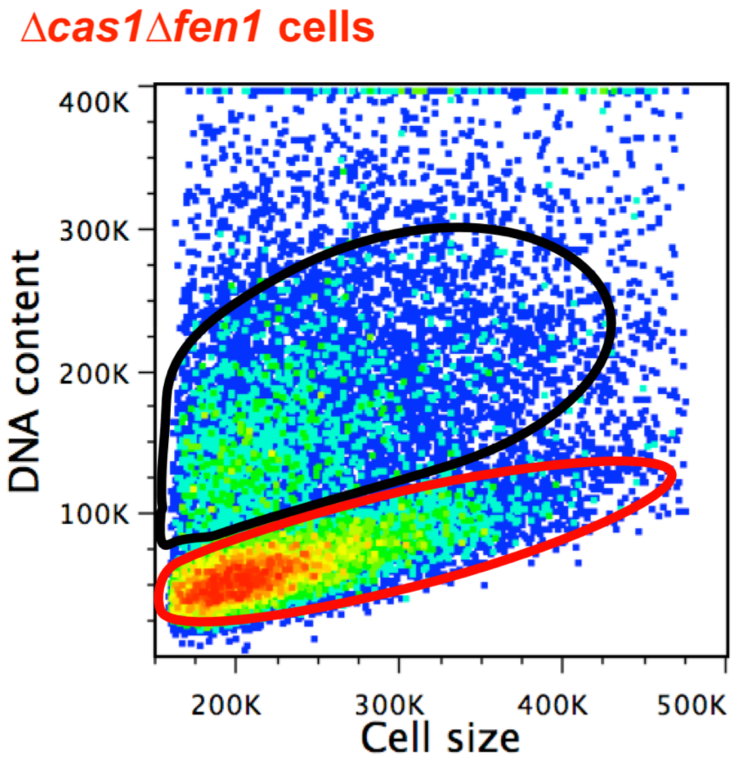

Supplementary Figure 2. Analysis of DNA content versus cell size. Plotting of DNA content versus cell size reveals two discrete populations for the Δ*cas1*Δ*fen1* mutant: a low DNA content population (63.5%) and a population with a DNA content comparable to wild-type cells (H119) (26.8%).

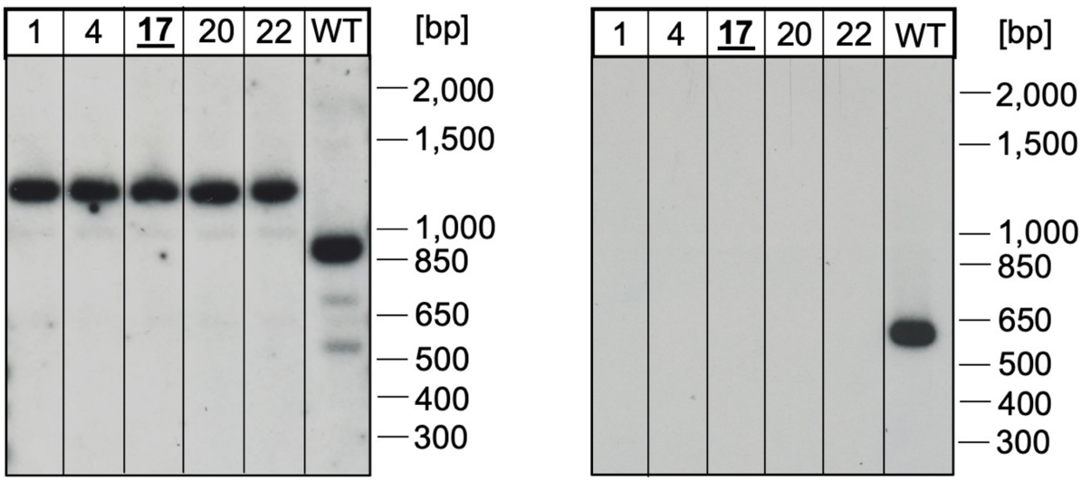

A. **B.**

Supplementary Figure 3. Southern blot analysis of Δ*cas1*Δ*fen1* clones. Chromosomal DNAs of Δ*cas1*Δ*fen1* pop-out candidates K1, K4, K17, K20, K22 (lanes 1,4,17,20 and 22) and wild-type strain H119 (WT) were extracted and digested by *Sal*I. A Southern blot analysis was made using two radioactively labelled probes to verify the deletion of the *fen1* gene in the Δ*cas1* strain. **A.** The membrane was hybridised with a probe binding upstream of the *fen1* gene. For the wild-type genome a signal at 920 bp, for Δ*fen1* mutants a signal at 1,210 bp was observed as expected. **B.** The membrane was hybridised with a *fen1* gene specific probe which only binds if *fen1* is not deleted. The expected signal would be at 570 bp as seen in the wild-type genome. All pop-out candidates investigated were indeed Δ*cas1*Δ*fen1* mutants. Clone 17 was selected for further experiments.

References

Allers, T., S. Barak, S. Liddell, K. Wardell, and M. Mevarech. 2010. 'Improved strains and plasmid vectors for conditional overexpression of His-tagged proteins in Haloferax volcanii', *Appl Environ Microbiol*, 76: 1759-69.

Allers, T., H. P. Ngo, M. Mevarech, and R. G. Lloyd. 2004. 'Development of additional selectable markers for the halophilic archaeon Haloferax volcanii based on the leuB and trpA genes', *Appl Environ Microbiol*, 70: 943-53.

Babu, M., N. Beloglazova, R. Flick, C. Graham, T. Skarina, B. Nocek, A. Gagarinova, O. Pogoutse, G. Brown, A. Binkowski, S. Phanse, A. Joachimiak, E. V. Koonin, A. Savchenko, A. Emili, J. Greenblatt, A. M. Edwards, and A. F. Yakunin. 2011. 'A dual function of the CRISPR-Cas system in bacterial antivirus immunity and DNA repair', *Mol Microbiol*, 79: 484-502.

Dierolf, J. 2010. 'Charakterisierung des CRISPR/Cas Systems in Haloferax volcanii', Ulm University, Germany.

Fischer, Susan. 2009. 'Charakterisierung von Proteinen des RNA-Metabolismus in Haloferax volcanii', Ulm University.

Goujon, M., H. McWilliam, W. Li, F. Valentin, S. Squizzato, J. Paern, and R. Lopez. 2010. 'A new bioinformatics analysis tools framework at EMBL-EBI', *Nucleic Acids Res*, 38: W695-9.

Kim, T. Y., M. Shin, L. Huynh Thi Yen, and J. S. Kim. 2013. 'Crystal structure of Cas1 from Archaeoglobus fulgidus and characterization of its nucleolytic activity', *Biochem Biophys Res Commun*, 441: 720-5.

Klein, C. 2011. 'Die Untersuchung des CRISPR/Cas Systems in Haloferax volcanii', Ulm University.

Lestini, R., Z. Duan, and T. Allers. 2010. 'The archaeal Xpf/Mus81/FANCM homolog Hef and the Holliday junction resolvase Hjc define alternative pathways that are essential for cell viability in Haloferax volcanii', *DNA Repair (Amst).* 9: 994-1002. doi: 10.16/j.dnarep.2010.06.012. Epub 10 Jul 27.

McWilliam, H., W. Li, M. Uludag, S. Squizzato, Y. M. Park, N. Buso, A. P. Cowley, and R. Lopez. 2013. 'Analysis Tool Web Services from the EMBL-EBI', *Nucleic Acids Res*, 41: W597-600.

Meslet-Cladiére, L., C. Norais, J. Kuhn, J. Briffotaux, J. W. Sloostra, E. Ferrari, U. Hübscher, D. Flament, and H. Myllykallio. 2007. 'A novel proteomic approach identifies new interaction partners for proliferating cell nuclear antigen', *J Mol Biol*, 372: 1137-48.

Rollie, Clare, Stefanie Schneider, Anna Sophie Brinkmann, Edward L. Bolt, and Malcolm F. White. 2015. 'Intrinsic sequence specificity of the Cas1 integrase directs new spacer acquisition', *eLife*, 4: e08716.

Sievers, F., A. Wilm, D. Dineen, T. J. Gibson, K. Karplus, W. Li, R. Lopez, H. McWilliam, M. Remmert, J. Söding, J. D. Thompson, and D. G. Higgins. 2011. 'Fast, scalable generation of high-quality protein multiple sequence alignments using Clustal Omega', *Mol Syst Biol*, 7: 539.
